# Phosphoproteomics revealed cellular signals immediately responding to disruption of cancer amino acid homeostasis induced by inhibition of l-type amino acid transporter 1

**DOI:** 10.1186/s40170-022-00295-8

**Published:** 2022-11-10

**Authors:** Hiroki Okanishi, Ryuichi Ohgaki, Minhui Xu, Hitoshi Endou, Yoshikatsu Kanai

**Affiliations:** 1 Department of Bio-system Pharmacology, Graduate School of Medicine, Osaka University, Osaka, Japan; 2 Integrated Frontier Research for Medical Science Division, Institute for Open and Transdisciplinary Research Initiatives (OTRI), Osaka University, Osaka, Japan; 3 J-Pharma Co., Ltd., Yokohama, Japan

**Keywords:** Amino acid transporter system, Drug combinations, Neoplasms, Proteomics

## Abstract

**Background:**

Cancer-upregulated l-type amino acid transporter 1 (LAT1; SLC7A5) supplies essential amino acids to cancer cells. LAT1 substrates are not only needed for cancer rapid growth, but involved in cellular signaling. LAT1 has been proposed as a potential target for cancer treatment—its inhibitor, JPH203, is currently in clinical trials and targets biliary tract cancer (BTC). Here, we revealed to what extent LAT1 inhibitor affects intracellular amino acid content and what kind of cellular signals are directly triggered by LAT1 inhibition.

**Methods:**

Liquid chromatography assay combined with *o*-phthalaldehyde- and 9-fluorenyl-methylchloroformate-based derivatization revealed changes in intracellular amino acid levels induced by LAT1 inhibition with JPH203 treatment in three BTC cell lines. Tandem mass tag-based quantitative phosphoproteomics characterized the effect of JPH203 treatment on BTC cells, and suggested key regulators in LAT1-inhibited cells. We further studied one of the key regulators, CK2 protein kinase, by using Western blot, enzymatic activity assay, and co-immunoprecipitation. We evaluated anticancer effects of combination of JPH203 with CK2 inhibitor using cell growth and would healing assay.

**Results:**

JPH203 treatment decreased intracellular levels of LAT1 substrates including essential amino acids of three BTC cell lines, immediately and drastically. We also found levels of some of these amino acids were partially recovered after longer-time treatment. Therefore, we performed phosphoproteomics with short-time JPH203 treatment prior to the cellular compensatory response, and revealed hundreds of differentially phosphorylated sites. Commonly downregulated phosphorylation sites were found on proteins involved in the cell cycle and RNA splicing. Our phosphoproteomics also suggested key regulators immediately responding to LAT1 inhibition. Focusing on one of these regulators, protein kinase CK2, we revealed LAT1 inhibition decreased phosphorylation of CK2 substrate without changing CK2 enzymatic activity. Furthermore, LAT1 inhibition abolished interaction between CK2 and its regulatory protein NOLC1, which suggests regulatory mechanism of CK2 substrate protein specificity controlled by LAT1 inhibition. Moreover, we revealed that the combination of JPH203 with CK2 inhibitor resulted in the enhanced inhibition of proliferation and migration of BTC cells.

**Conclusion:**

This study provides new perspectives on LAT1-dependent cellular processes and a rationale for therapeutics targeting reprogrammed cancer metabolism.

**Supplementary Information:**

The online version contains supplementary material available at 10.1186/s40170-022-00295-8.

## Background

Dysregulated cellular amino acid uptake is one of the hallmarks of cancer-associated metabolic changes [1]. Amino acids are the source of synthesis of three major macromolecules: proteins, nucleic acids, and lipids. Cancer cells produce these macromolecules necessary for their rapid growth. Their biosynthesis is highly dependent on the uptake of extracellular amino acids [2]. Amino acids are also known as cellular signaling regulators involved in various biological processes such as cell proliferation, survival, and cancer progression [3]. Specific transporters on the plasma membrane mediate the uptake of extracellular amino acids into cells. In cancer cells, amino acid transporters are generally upregulated and contribute to cancer cell growth and proliferation [3, 4].

l-type amino acid transporter 1 (LAT1), one of the system L amino acid transporters [4, 5], is highly expressed in various types of tumors, but scarcely in normal tissues [4]. Several clinical applications of LAT1 have been proposed including cancer-specific positron emission tomography [6, 7], cancer diagnostic and prognostic markers [8], and targeted therapies for cancer treatment [3, 9]. A LAT1-specific inhibitor, JPH203 (also called KYT-0353), was developed [10] and, in preclinical studies, exerted growth inhibitory effects on xenograft tumors from various types of cancers, including biliary tract cancer (BTC) [11], colon cancer [10], T cell lymphoblastic lymphoma/T cell acute lymphoblastic leukemia [12], thymic carcinoma [13, 14], and thyroid cancer [15]. In the first-in-human phase I clinical trial, JPH203 appeared to provide promising activity particularly against BTC [16]. These studies have highlighted LAT1 as a potential therapeutic target for cancers [4].

LAT1 not only supplies cancer cells with amino acids essential for biomolecule synthesis but also regulates phosphorylation signaling [3, 4]. LAT1 inhibition dramatically decreases the uptake of leucine, one of the LAT1 substrates, in cancer cells [8, 10, 11, 14, 15, 17–19], which implies that leucine uptake strongly depends on LAT1. Therefore, it has been well established that leucine stimulates the mechanistic target of rapamycin kinase complex 1 (mTORC1), known as a central hub of nutrient signaling [3, 20]. Consistent with this, previous studies have shown that LAT1 inhibition reduces mTORC1 activity [12, 15, 18, 21]. Therefore, it seems reasonable to assume that LAT1 continuously stimulates phosphorylation signals by maintaining intracellular amino acid pools in cancer cells. Elucidation of these LAT1-dependent signals would expand our understanding of cancer metabolic reprogramming, and provide candidates for LAT1 inhibitor-based combination therapies. To comprehensively examine cancer cellular signaling, phosphoproteomics is a powerful tool [22]. However, cellular homeostatic responses should be considered when examining phosphorylation changes induced by drugs which interrupt metabolic pathway. For example, the decrease of intracellular amino acid levels induces the transcription of amino acid transporters [23–25], and autophagy [25], which compensates for starved amino acids induced by LAT1 inhibition. Through these mechanisms, long-term LAT1 inhibition may lead to recovery of the decreased LAT1 substrate levels, and restore the altered cellular signaling. To avoid effects of compensatory metabolic processes, it may be useful to study phosphorylation changes with short-time drug treatment before homeostatic responses occur. However, no studies on nutrient flux-inhibited conditions have applied this approach to examine phosphoproteome, compared with long-time treatment.

Previously, we conducted phosphoproteomics on BTC cells treated with JPH203 for 24 h, and identified thousands of changes in phosphorylation [26]. The study provided insights into changes in signaling pathways that contribute to the anticancer effect of JPH203. However, as mentioned above, such prolonged LAT1 inhibition is inadequate for examining the alteration of phosphorylation signals directly caused by LAT1 inhibition because of possible homeostatic compensatory responses and feedback signal regulation. Therefore, in this study, we conducted phosphoproteomics on cancer cells subjected to short-time JPH203 treatment. We, moreover, measured the intracellular LAT1 substrate levels to evaluate phosphoproteomics data in immediate response to the intracellular amino acid level changes due to LAT1 inhibition. In addition, the downregulated phosphorylation of proteins involved in cell cycle regulation and RNA splicing, immediately following LAT1 inhibition, occurred commonly among cell lines. Furthermore, this study focused on a possible regulator revealed by phosphoproteomics, CK2, a highly active kinase in cancers, involved in various biological processes, including cell cycle regulation and RNA splicing [27–31]. We found downregulation of CK2-related pathways by LAT1 inhibition and enhanced suppression of BTC cell proliferation and migration by combining JPH203 and a CK2-inhibitor, suggesting a potential candidate for LAT1 inhibitor-based combination therapy.

## Methods

### Experimental design and statistical rationale

This study was designed to performed quantitative phosphoproteomics on the LAT1-inhibited cancer cells by JPH203 treatment. The sample size for each JPH203-treated and non-treated control sample consisted of three biological replicates (*n* = 3), which allowed evaluation of statistical comparison of fold change by Student’s *t* test. The comparison of phosphoproteome between JPH203-treated and non-treated samples were performed for biliary tract cancer cell lines (KKU-055, KKU-100, and KKU-213), respectively.

### Cell culture

Human BTC cell lines, KKU-055 (JCRB1551), KKU-100 (JCRB1568), and KKU-213 (JCRB1557), were provided by the Japanese Collection of Research Bioresources. RPMI-1640 and penicillin/streptomycin were purchased from Nacalai Tesque (Kyoto, Japan). Fetal bovine serum (FBS) was from Gibco (Thermo Fisher Scientific, Waltham, MA, USA). The cells were cultivated in RPMI-1640 supplemented with 10% FBS and penicillin/streptomycin at 37 °C with 5% CO_2_.

### Tryptic peptide preparation

The cells were seeded at optimized density depending on the cell lines: 1.4 × 10^4^ cells/cm^2^ cells/mL for KKU-055 and KKU-100, and 2.8 × 10^4^ cells/cm^2^ for KKU-213. Six hours before harvesting, the medium was changed with the fresh one. Forty-eight hours after the seeding, the cells were treated with or without 30 μM JPH203 (J-Pharma, Tokyo, Japan) with a final DMSO concentration of 0.3% for 15 or 30 min. Then, the cells were washed with ice-cold phosphate-buffered saline containing a complete protease inhibitor cocktail (Sigma-Aldrich, St. Louis, MO, USA) and PhosSTOP (Sigma-Aldrich), and harvested.

Proteomics sample preparation using a modified phase transfer surfactant-aided digestion strategy [32] was conducted as described previously [26]. Briefly, the cells were lysed in 100 mM Tris (pH 8.5) containing 12 mM sodium deoxycholate, 12 mM sodium N-lauroyl sarcosinate, and PhosSTOP by heating. After reduction with dithiothreitol and following alkylation with iodoacetamide, the protein samples were diluted 5-fold with 50 mM ammonium bicarbonate. The proteins were digested with lysyl endopeptidase and trypsin until enough digestion. The resultant solution was desalted by a Sep-Pak Plus C_18_ cartridge (Waters, Milford, MA, USA).

### Phosphoproteomics sample preparation

Phosphopeptide enrichment from tryptic digests by IMAC, TMT labeling, and fractionation was conducted as previously described [26]. In short, immobilized Fe(III) affinity chromatography (Fe(III)-IMAC) resin was made from nickel-chelating resin. After chelated Ni^2+^ ion was released from resin with 50 mM EDTA, Fe^3+^ ion was chelated to the resin. The resultant Fe(III) IMAC resin was equilibrated with 0.1% TFA/60% acetonitrile. Then, tryptic peptides were loaded into the resin. The resin was washed with 0.1% TFA/60% acetonitrile and subsequently washed with 0.1% TFA. Then, the enriched phosphopeptides were eluted with 1% phosphoric acid.

Phosphopeptides were desalted by C18 StageTip method using 3M Empore C18 disk (GL Science, Tokyo, Japan). After dried up, phosphopeptides were labeled with TMT 10-plex isobaric label reagent (Thermo Fisher Scientific, Waltham, MA, USA) according to the manufacturer’s protocol (Table S1). TMT-labeled phosphopeptides were mixed, followed by high pH C18 fractionation.

### LC-MS/MS analysis, data analysis, and in silico analysis

Fractionated phosphopeptides were analyzed by LC-MS/MS as previously described [26]. The peptides of each sample were separated on a nano HPLC system. The eluted peptides were analyzed with a Q-Exactive orbitrap mass spectrometer (Thermo Fisher Scientific), and MS and MS/MS spectral data were acquired.

Phosphopeptides were identified with the threshold of *q* value 0.01 by analyzing MS and MS/MS data using Proteome Discoverer 2.3 software (Thermo Fisher Scientific). As suggested in the previous study of quantitative proteomics by quantitation at the MS/MS level using TMT in which multiple testing corrections fail to detect true positives [33], a *p* value cut-off and an effect size cut-off were used to define differentially phosphorylated sites as follows; *p* value < 0.05 and fold change ≥ 1.2. Differentially phosphorylated sites were analyzed by Ingenuity Pathway Analysis (IPA; QIAGEN, Hilden, Germany) to detect activated and inactivated pathways and to suggest upstream regulators.

Functional annotation of proteins with differentially upregulated and downregulated phosphorylation sites was analyzed by GO analysis using DAVID (v6.8) [34], respectively. The GO FAT terms were clustered, and the clusters with enrichment score > 2 were extracted. The top terms in the clusters were listed. Networks of proteins with differentially upregulated and downregulated phosphorylation sites were generated by STRING (version 11.0) [35], respectively.

### Western blotting

Western blotting was conducted as previously described [26]. In Western blotting, the following antibodies were used at the indicated dilutions: p70 S6K–pT389 (1:1000, #9234), p70 S6K (1:1000, #9202), phospho–CK2 substrate (1:1000, #8738) from Cell Signaling Technology (Danvers, MA, USA); CK2β (1:500, 22418-1-AP), NOLC1 (1:1000, 11815-1-AP), and β-actin (1:5000, 66009-1-Ig) from Proteintech Group (Chicago, IL, USA); TOP2A-pS1469 (1:1000, PA5-64536) from Affinity Biosciences (Cincinnati, OH, USA); CK2β–pS209 (1:1000, 44-1090G) from Invitrogen (Waltham, MA, USA); TOP2A (1:200, sc-365916), CK2α (1:1000, sc-373894) from Santa Cruz Biotechnology (Dallas, TX, USA); goat anti-mouse IgG-HRP (1:5000, 115-035-062), goat anti-rabbit IgG-HRP (1:5000, 111-035-003) from Jackson ImmunoResearch Laboratories (West Grove, PA, USA).

### HPLC analysis of intracellular amino acids

The cells were seeded on 6-well plate at the same densities that was used for phosphoproteomics. Six hours before harvesting, the medium was changed with the fresh one. Forty-eight hours after the seeding, the cells were treated with 30 μM JPH203 with final DMSO concentration of 0.3%. After treatment for the indicated time, the cells were washed twice with cold PBS, followed by three freeze-thaw cycle in 1 mL of water on ice. After centrifuged at 15,000×*g* at 4 °C for 10 min, 800 μL of the supernatant was mixed with 800 μL of acetonitrile. In addition, 20 μL of the supernatant was used for BCA assay. The extract was centrifuged at 15,000×*g* at 4 °C for 10 min. Then, 500 μL of the supernatant was harvested, and dried up by a centrifugal evaporator CC-105 (TOMY, Tokyo, Japan). The resultant amino acids were analyzed by 1260 Infinity II LC System (Agilent Technologies, CA, USA). Each sample was injected via the autosampler, and was reacted with 9-fluorenylmethylchloroformate reagent (FMOC; Agilent Technologies) and *o*-phthalaldehyde/3-mercaptopropionic acid reagent (OPA; Agilent Technologies) in sample loop for derivatization according to manufacturer’s protocol. The fluorescent amino acid derivatives loaded to a Poroshell HPH-C18 (3.0 × 5 mm, 2.7 μm resin size; Agilent Technologies) and Poroshell HPH-C18 (3.0 × 100 mm 2.7 μm resin size; Agilent Technologies) with solvent A (20 mM Na_2_HPO_4_, pH 8.2), and were separated at a flow rate of 0.65 mL/min on the column at 40 °C with the following gradient; 4% solvent B (methanol:acetonitrile:H_2_O = 45:45:10) for 0 min; 4–57% solvent B for 13 min; 57–100% solvent B for 1 min. The fluorescence detector was set to an excitation wavelength of 230 nm and an emission wavelength of 450 nm for detecting OPA-derivatives and changed to an excitation wavelength of 266 nm and an emission wavelength of 305 nm at 12 min for detecting FMOC-derivatives. The standard amino acids were also analyzed by HPLC. We added asparagine, glutamine, and tryptophan (Sigma-Aldrich, MO, USA) to amino acid standard containing the other standard 17 amino acids (Agilent Technologies) and used them to make standard curve for quantification.

### Cell growth inhibition assay

The cells of 1.0 × 10^3^ were cultured on 96-well plate for 24 h in 100 μL of the culture medium. The cells were, then, treated with JPH203 and/or CX-4945 (Monmouth Junction, NJ, USA) at the indicated concentration in the culture medium with final DMSO concentration of 0.3%. In the combination assay, the concentration of inhibitors used are as follows: 1.1 μM JPH203, 3.8 μM CX-4945 for KKU-055; 12 μM JPH203, 4.5 μM CX-4945 for KKU-100; 12 μM JPH203, 2.5 μM CX-4945 for KKU-213. After the treatment with inhibitors for 3 days, the cell growth was assessed by water-soluble tetrazolium salt (WST) assay using Cell Counting Kit-8 (Dojindo, Kumamoto, Japan). The absorbance at 450 nm, relevant to cell number, with JPH203 treatment was expressed as % of the control without JPH203 treatment.

### Cell migration inhibition assay

The cells of 2.8 × 10^4^ in 70 μL of the medium were cultured on 2-well silicone culture insert (ibidi, Bavaria, Germany) settled in 24-well plate for 24 h. The insert was removed, and the cells were washed with PBS at 37 °C. Then, cell migration was initiated in the medium containing with 30 μM JPH203 and/or 5 μM CX-4945 with final DMSO concentration of 0.8%. After 24 h cultivation for migration, cells were washed with PBS, followed by staining with 0.25% crystal violet in 20% methanol for 10 min. After washed with PBS five times, the migrated cells were subjected to image acquisition using an inverted microscope CMi1 (Leica Microsystems, Wetzlar, Germany). The number of migrated cells with inhibitor treatment was counted by using ImageJ software with Cell Counter plugin (NIH), and expressed as % of the control without inhibitor treatment.

### CK2 activity assay

The cells treated with 30 μM JPH203 for 2 h and 24 h, and the control cells, were washed with PBS twice, and harvested, respectively. The cells were lysed in 50 mM Tris (pH 7.4) containing 150 mM NaCl, 1% Triton X-100, and complete protease inhibitor cocktail (Sigma–Aldrich). The extracted protein samples were diluted, and 10 μg protein in 10 μL was used for CK2 activity assay by using CycLex CK2 Kinase Assay/Inhibitor Screening Kit (MBL, Woburn, MA, USA) according to manufacturer’s protocol. Phosphorylation of p53 N-terminal peptide, CK2 substrate, coated to 96 wells was detected by ELISA using p53-pS46 antibody conjugated with horseradish peroxidase.

### Co-immunoprecipitation

The control cells and the cells treated with 30 μM JPH203 for 24 h were lysed in 50 mM Tris (pH 7.4) containing 150 mM NaCl, 1% Triton X-100, PhosSTOP and cOmplete protease inhibitor cocktail. The extracted protein sample was diluted, and 300 μg of protein in 300 μL was mixed with 1 μg of anti-NOLC1 antibody. The sample was rotated at 4 °C for 1 h, followed by incubation with 20 μL of protein A-agarose beads (Santa Cruz Biotechnology, Dallas, TX, USA) for 1 h. After washing with PBS, the beads were boiled in laemmli sample buffer. The eluate was subject to SDS-PAGE and following Western blot.

### Data accessibility

The MS raw data, peak list files, and result files were deposited in the ProteomeXchange Consortium (http://www.proteomexchange.org/; PXID, PXD034276) via the jPOST partner repository (https://jpostdb.org; jpost ID, JPST001655).

## Results

### The effect of LAT1 inhibition on the levels of intracellular LAT1 substrates

**Fig. 1:**
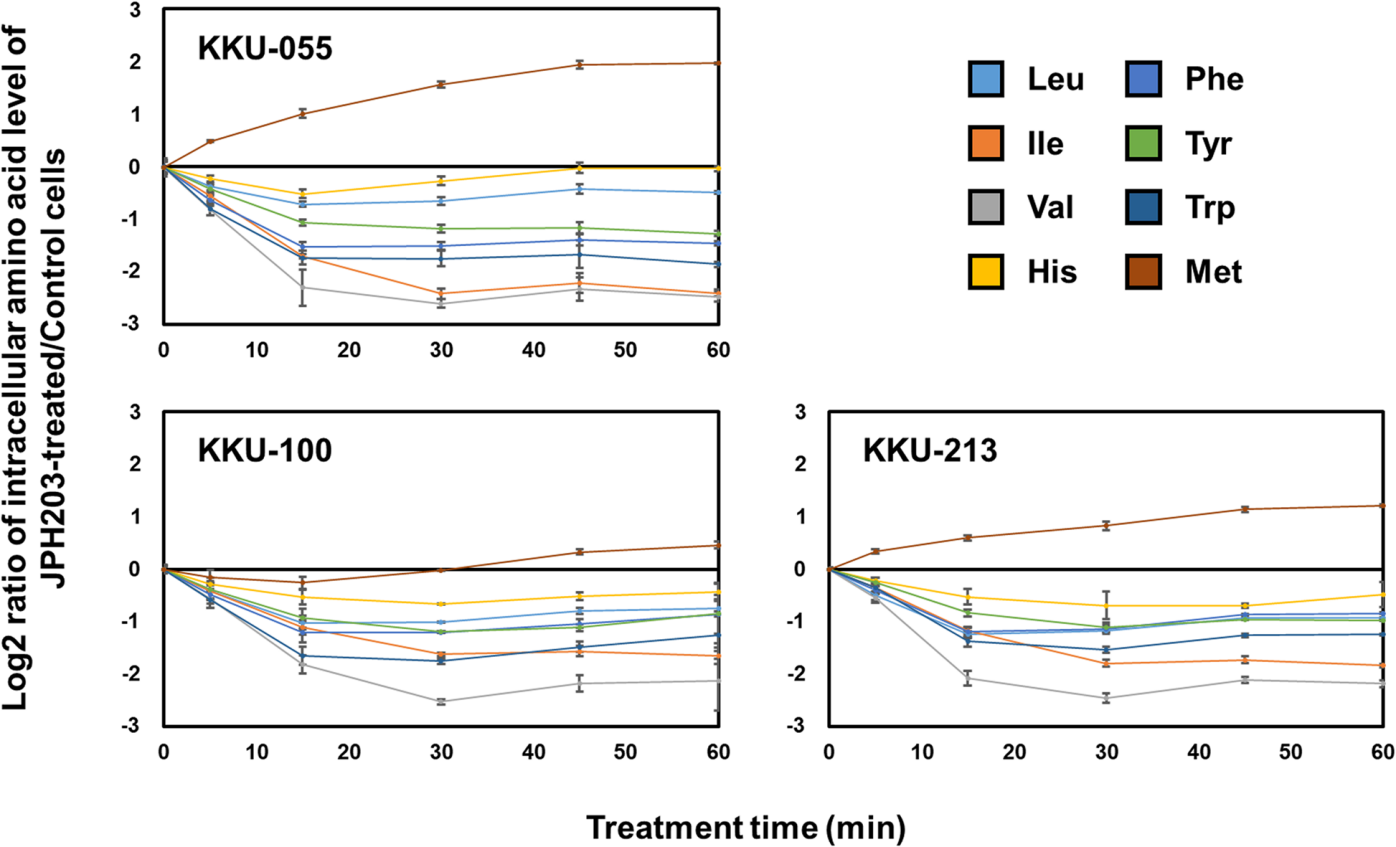
Decreased intracellular amino acid level by JPH203 treatment in BTC cells. Log2 ratio of the intracellular amino acid level of JPH203-treated cells vs. control cells is plotted for each amino acid against the time (min) after starting JPH203 treatment. Each amino acid amount was normalized by protein amount (pmol/μg protein). Data represent means ± SD (*n* = 3)

To reveal phosphorylation signals that depend on the intracellular amino acids maintained by LAT1 in cancer cells, we performed phosphoproteomics on cancer cells subjected to pharmacologic LAT1 inhibition. To determine the optimal treatment time, we investigated the time course of intracellular amino acid levels after starting the treatment with a selective LAT1 inhibitor, JPH203. Because the phase I clinical trial suggested a clinical benefit of JPH203 to treat patients with BTC [16], we selected three BTC cell lines (KKU-055, KKU-100, and KKU-213) for the following analysis. Combining *o*-phthalaldehyde- and 9-fluorenyl-methylchloroformate-based derivatization with LC separation to measure 20 normalized standard amino acids, 19 amino acids except cysteine were detected in the BTC samples (Fig. 1). In all three cell lines tested, treatment with JPH203 for 5 min reduced seven of the eight LAT1 substrates: leucine, isoleucine, valine, phenylalanine, tyrosine, tryptophan, and histidine (excluding methionine). Levels of these seven amino acids were further reduced in all three cell lines 15 and 30 min after JPH203 treatment. The levels of decrease at 15 min were 38.7 to 57.8% for leucine, 53.2 to 69.0% for isoleucine, and 71.3% for valine. 56.2 to 65.0% for phenylalanine, 43.9 to 51.9% for tyrosine, 61.3 to 69.7% for tryptophan, and 29.4 to 30.7% for histidine, whereas those at 30 min were 35.7 to 55.6% for leucine, 67.4 to 81.0% for isoleucine, 81.8 to 83.3% for valine, 54.5 to 64.7% for phenylalanine, 53.5 to 55.9% for tyrosine, 65.4 to 70.3% for tryptophan, and 16.2 to 37.5% for histidine (Fig. 1). These observations demonstrate a high dependence of seven amino acid levels on LAT1-mediated uptake. After longer treatments (45 and 60 min), levels of some of these amino acids, such as histidine, were partially recovered (Fig. 1). Furthermore, short-time JPH203 treatment (15 and 30 min) did not commonly reduce levels of non-LAT1 substrate amino acids and a LAT1 substrate, methionine (Figure S1). Based on these results, we selected a 15- and 30-min treatment of JPH203 for the following phosphoproteome analysis to examine the phosphorylation signals directly dependent on the intracellular amino acids maintained by LAT1 without being affected by the compensatory mechanism of the cells.

### Identification of differentially phosphorylated sites between LAT1-inhibited and control BTC cells

**Fig. 2:**
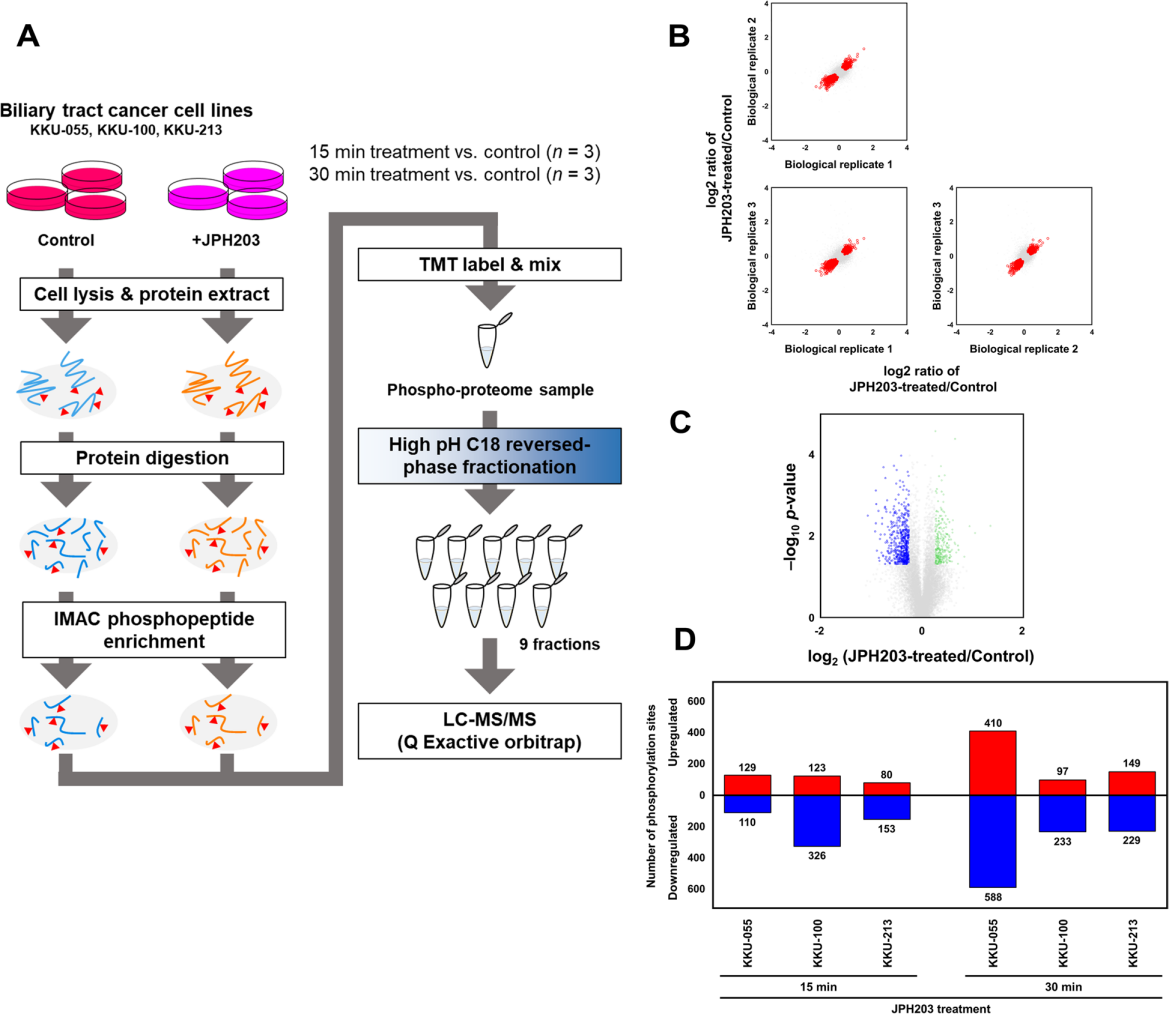
Phosphoproteomics of BTC cell lines subjected to short-time JPH203 treatment. **A** Quantitative phosphoproteomics workflow for studying the effects of short-time LAT1 inhibition on BTC cells. Tryptic peptide samples were prepared from cells treated with 30 μM JPH203 for 15 and 30 min, and the control non-treated cells (*n* = 3). Phosphopeptides were enriched by IMAC. After being labeled with TMT reagent and mixed, phosphopeptide samples were subject to high pH C18 fractionation. All fractions were analyzed by Q-Exactive mass spectrometer. **B** Representative scatter plots showing log2 fold changes of phosphoproteome between biological replicates of JPH203-treated/control samples of KKU-055 cells treated with JPH203 for 30 min. Plots of differentially phosphorylated sites are shown in red. **C** Representative phosphoproteomics data from JPH203-treated and control samples of KKU-055 cells treated with JPH203 for 30 min. A volcano plot was generated by plotting -log10 *p* values against log2 fold changes of relative abundance ratio between JPH203-treated/control samples. Plots of differentially phosphorylated sites are shown in green and blue. **D** The number of differentially phosphorylated sites in cells subjected to short-time LAT1 inhibition. The bars of upregulated and downregulated phosphorylation sites are shown in red and blue, respectively

We conducted global phosphoproteomics on three BTC cell lines treated with JPH203 for 15 min and 30 min (Fig. 2A). High pH C18 StageTip method fractionated phosphopeptides enriched by IMAC from trypsin digestion peptides to increase the number of identified peptides, followed by LC-MS/MS with MS2-based TMT quantification. We identified an average of 20,688 quantifiable phosphopeptides (Table S2) and 15,336 phosphorylation sites (Table S3). Furthermore, MS2-based TMT quantification provided the differentially phosphorylated sites between JPH203-treated and control cells in three BTC cell lines (Fig. 2B, C, S2 and S3). We revealed differentially phosphorylated sites of BTC cells treated with JPH203 for 15 min: 239, 449, and 233 sites in KKU-055, KKU-100, and KKU-213, respectively (Fig. 2D). These included 129, 123, and 80 upregulated and 110, 326, and 153 downregulated sites in KKU-055, KKU-100, and KKU-213. Furthermore, our phosphoproteomics showed differentially phosphorylated sites in BTC cells treated with JPH203 for 30 min: 998, 330, and 378 sites in KKU-055, KKU-100, and KKU-213, respectively (Fig. 2D). Of these, 410, 97, and 149 upregulated and 588, 233, and 229 downregulated sites were found in KKU-055, KKU-100, and KKU-213.

### Upstream regulator analysis and pathway analysis of differentially phosphorylated sites

**Fig. 3:**
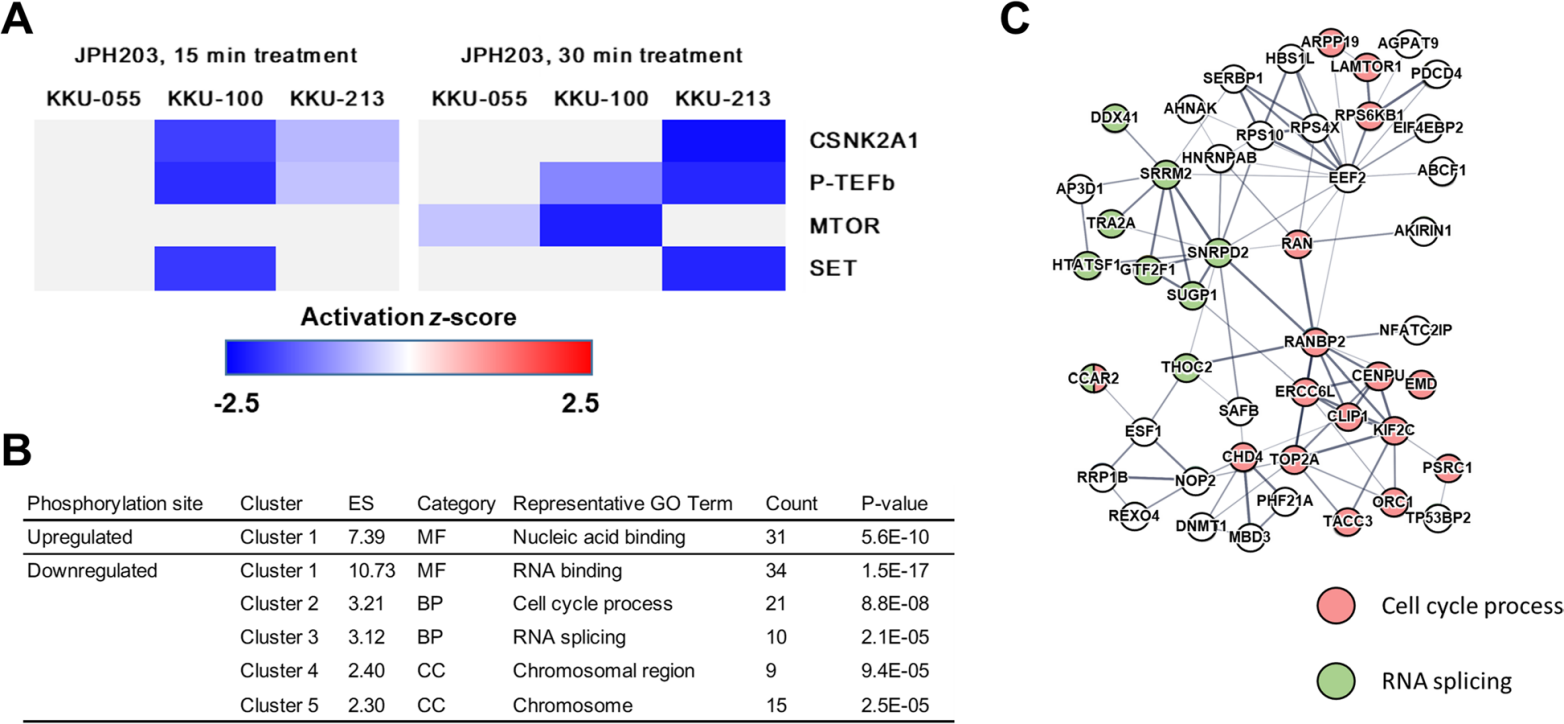
Possible regulators and functions responding to short-time LAT1 inhibition. **A** Heatmap showing activation and inactivation of possible regulators commonly detected in at least two cell lines. Activation *z*-scores of activated or inactivated regulators from phosphoproteomics of KKU-055, KKU-100, and KKU-213 are shown by IPA. **B** Functional annotation clustering using DAVID for proteins with upregulated and downregulated phosphorylation commonly detected in at least two phosphoproteome results. The table shows the top terms of each cluster. ES, enrichment score; MF, molecular function; BP, biological process; CC, cellular component. **C** A network of proteins with downregulated phosphorylation commonly detected in at least two phosphoproteomics results. Proteins categorized into two biological processes in DAVID analysis in Fig. 3B, cell cycle process, and RNA splicing, are shown in magenta and green, respectively

To investigate how LAT1 inhibition by short-time JPH203 treatment affects phosphorylation regulators, upstream regulator analysis was performed using IPA software. Upstream regulator analysis of proteins with differentially phosphorylated sites showed 8 and 3 possible regulators in KKU-100 and KKU-213 treated with JPH203 for 15 min, respectively, and 21, 12, and 5 possible regulators in KKU-055, KKU-100, and KKU-213 treated with JPH203 for 30 min, respectively (Table S4). Of these, four regulators were commonly downregulated in at least two cell lines: three kinases and a phosphatase inhibitor (Fig. 3A and Table S5), which were not found in the previous phosphoproteomics study on 24 h JPH203 treatment [26]. Inactivation of three kinases was predicted: CSNK2A1 (α subunit of CK2 kinase) in KKU-100 and KKU-213, P-TEFb (transcription elongation complex regulating CDK9 and cyclin T) in KKU-100 and KKU-213, and mTOR in KKU-055 and KKU-100. Inactivation of a phosphatase inhibitor, SET (an inhibitor of PP2A phosphatase), was also predicted in KKU-100 and KKU-213. Furthermore, the upstream regulator analysis suggested a decrease in amino acids and leucine in KKU-100 (Table S4), consistent with observed changes in intracellular amino acids (Fig. 1). In addition, possible inactivation of cell cycle-related kinases was shown in KKU-055: CDK1, CDK4, CDK6, CCNB1, CCNE1, and AURKA (Table S4). This is consistent with our previous study showing the influence of LAT1 inhibition on the cell cycle, especially CDKs-related pathways [26].

Furthermore, pathway analysis evaluated the roles of proteins with differentially phosphorylated sites. Proteins with differentially phosphorylated sites in each cell line were mapped to pathways using IPA and enriched in 32 pathways (Table S6). They included cell cycle-related pathways, including activation of “G1/S Checkpoint Regulation” and inactivation of “Cell Cycle Control of Chromosomal Replication” in KKU-055 treated with JPH203 for 30 min. Inactivation of “mTOR Signaling” and the activation of “PTEN Signaling” were found in KKU-100 treated with JPH203 for 30 min. Previously, we found that JPH203 treatment induces cell cycle arrest and suppresses growth in BTC cells [26]. Our present study suggests that the process of cell growth suppression occurs immediately after LAT1 inhibition.

### Analysis of commonly upregulated or downregulated phosphorylation sites

To further investigate the biological functions affected by short-time LAT1 inhibition, we focused on phosphorylation sites commonly upregulated or downregulated in at least two phosphoproteome results among six phosphoproteome results from three BTC cell lines treated with JPH203 for 15 min or 30 min (Table S7). GO analysis of proteins with commonly downregulated phosphorylation sites highlighted two biological processes (Fig. 3B and Table S8), “Cell cycle process (Cluster 2)” and “RNA splicing (Cluster 3)”, indicating that LAT1 inhibition generally decreased the phosphorylation of proteins involved in these processes. In addition, we further analyzed these commonly upregulated or downregulated phosphorylation sites by STRING and generated networks of proteins with the commonly changed phosphorylation sites (Figure S4). In the network, the “cell cycle process” includes proteins such as TOP2A, RPS6KB1, LAMTOR1, KIF2C, ORC1, and ERCC6L, whereas “RNA splicing” involves TRA2A, SNRPD2, and THOC2 (Fig. 3C). In addition, GO analysis also showed that phosphorylation of proteins involved in “RNA binding (Cluster 1)” and “chromosome (Clusters 4 and 5)” was commonly changed by LAT1 inhibition (Fig. 3B).

### Validation of phosphorylation decreased by short-time LAT1 inhibition

**Fig. 4:**
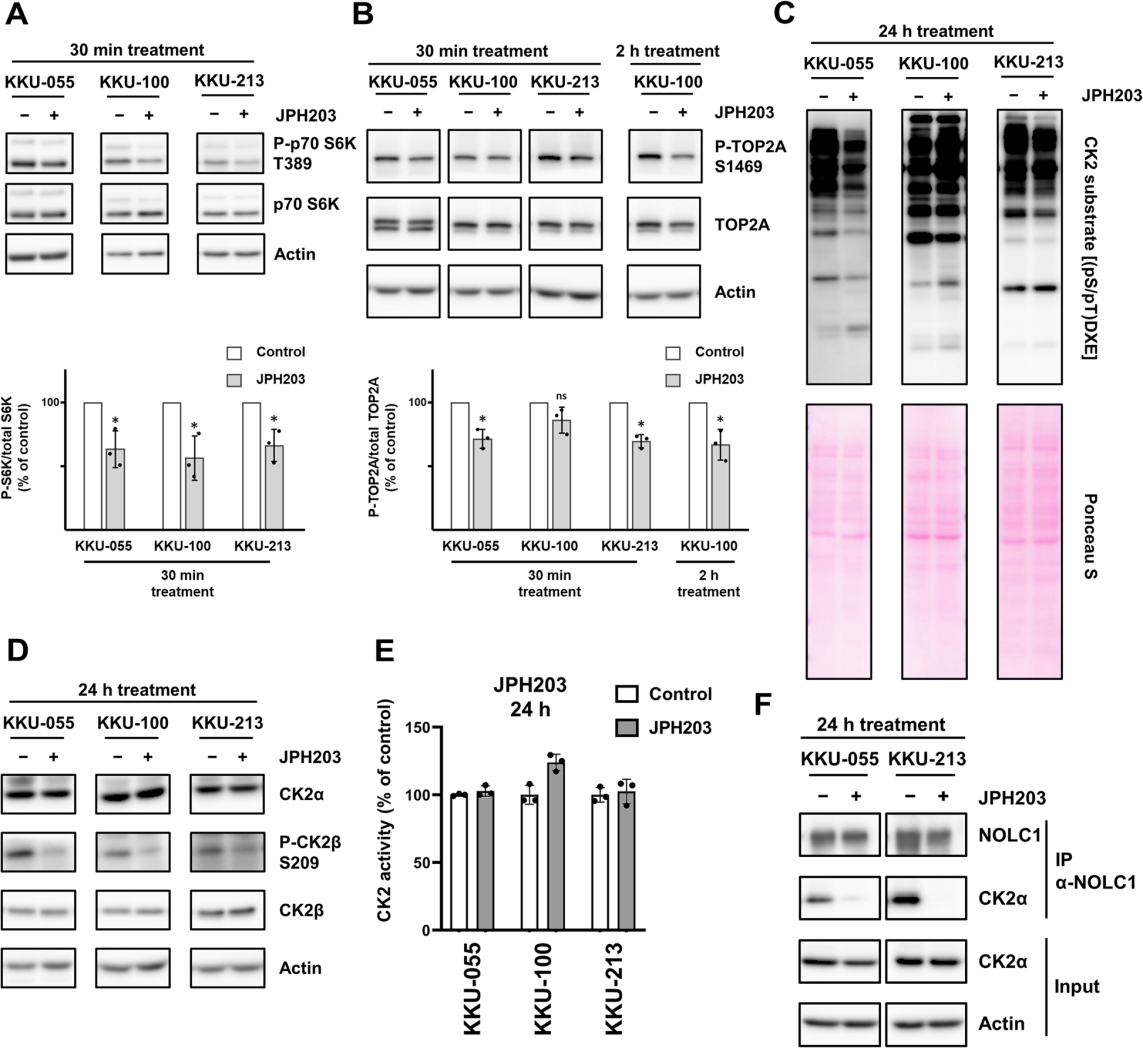
Changes in the phosphorylation of substrates of suggested key kinases and their regulatory proteins by LAT1 inhibition. Protein extracted from cells treated with 30 μM JPH203 and control cells was analyzed by Western blot. (A and B) The decrease in phosphorylation associated with JPH203 treatment for indicated time: Thr-389 of p70 S6K (**A**) and Ser-1469 of TOP2A (**B**). The data are presented as a ratio of the signal intensity of phosphorylated protein to total protein ± SD (*n* = 3). *p value < 0.05. ns = not significant (one sample *t* test) (**C**) Decreased phosphorylation of part of CK2 substrates by LAT1 inhibition with JPH203 for 24 h. Phosphorylation on the consensus CK2 substrate motif was detected by a specific antibody. **D** Decreased phosphorylation of Ser-209 of CK2β, a regulatory subunit of CK2, in cells treated with JPH203 for 24 h. **E** CK2 activity of BTC cells treated with JPH203. The extracted protein of BTC cells treated with 30 μM JPH203 for 24 h was subject to CK2 activity assay by ELISA using p53 N-terminal peptide and p53-pS46 antibody conjugated with horseradish peroxidase. **F** The decrease of CK2α co-immunoprecipitated with NOLC1. Protein extracted from KKU-055 and KKU-213 cells treated with JPH203 for 24 h was subject to immunoprecipitation using anti-NOLC1 antibody

Upstream regulator analysis indicated possible inactivation of mTOR (Fig. 3A), which is consistent with the decrease by JPH203 in intracellular leucine that stimulates mTORC1. Western blot confirmed that Thr-389 phosphorylation of the mTOC1 substrate p70 S6 kinase decreased in the three tested cell lines 30 min after JPH203 treatment (Fig. 4A), which supports our phosphoproteomics results.

Furthermore, we investigated key regulators of phosphorylation associated with LAT1 inhibition. Since our phosphoproteomics suggested cell cycle-related pathways and RNA splicing affected by short-time LAT1 inhibition (Fig. 3B, C), we focused on one of the common regulators involved in both biological processes: CK2α (CSNK2A1, Fig. 3A), a catalytic subunit of protein kinase CK2 [27–31]. Among the cell cycle-related proteins whose phosphorylation sites were commonly decreased by short-time LAT1 inhibition (Fig. 3C), we found TOP2A as a CK2 substrate based on PhosphoSitePlus kinase substrate database and previous studies [36, 37]. Western blot showed that phosphorylation at Ser-1469 of TOP2A, the CK2 substrate site, was decreased in KKU-055 and KKU-213 within 30 min, and in KKU-100 within 2 h after JPH203 treatment (Fig. 4B). In addition, we confirmed this effect by using another inhibitor of l-type amino acid transporters, 2-amino-2-norbornane carboxylic acid (BCH). BCH treatment also decreased phosphorylation at Ser-1469 of TOP2A (Figure S5). To further investigate the effect of LAT1 inhibition on CK2-mediated phosphorylation, we conducted Western blots using an antibody that recognizes phosphorylation on the CK2 substrate consensus sequence. After short-time treatment with JPH203, remarkable changes in the phosphorylation of CK2 substrates were not detectable by Western blot (Figure S6). The apparent discrepancy in the downregulation of CK2 phosphorylation between phosphoproteomics and Western blots may be due to different detection methods. Suppose phosphorylations of some of the many CK2 substrates are decreased by short-time JPH203 treatment; they can be detected by phosphoproteomics, whereas in Western blots, they may be hidden by a thick band of unchanged phosphorylation CK2 substrates. On the other hand, at 24 h of JPH203 treatment, when the phosphorylation of CK2 substrates becomes advanced more, the reduction of phosphorylation was detected in some bands of CK2 substrates in the Western blot of three cell lines (Fig. 4C), which confirmed the effect of LAT1 inhibition on CK2-mediated phosphorylation.

Because phosphorylation of not every CK2 substrate was decreased in 24 h JPH203 treatment (Fig. 4C), it would be reasonable to suppose that LAT1 inhibition may alter selectivity for substrate proteins rather than reduce CK2 activity. Interestingly, we found JPH203 treatment had no influence on the expression of catalytic CK2α (Fig. 4D) and did not reduce enzymatic activity against peptide substrate (Fig. 4E and Figure S7). To further provide clues to understanding CK2 regulation in LAT1-inhibited cells, we reanalyzed previous phosphoproteomics and proteomics results in cells treated with JPH203 for 24 h [26]. We investigated changes in expression and phosphorylation of previously reported CK2 regulatory proteins: CK2β (regulatory subunit of CK2) [38], NOLC1 (human Nopp140) [39, 40], and Pin1 [36], and found downregulated phosphorylation of Ser-209 of CK2β and several sites of NOLC1 in at least two cell lines (Table S9). In addition, expression of Pin1 was decreased in KKU-100 (Table S9).

To further investigate the mechanism of CK2 regulation by LAT1 inhibition, we performed co-immunoprecipitation assay using an anti-NOLC1 antibody. CK2α co-immunoprecipitated with its regulatory protein NOLC1 under the control condition, whereas JPH203 treatment drastically reduced co-immunoprecipitated CK2α (Fig. 4F), indicating that CK2-NOLC1 interaction is abolished by LAT1 inhibition. Furthermore, phosphorylation of CK2β at Ser-209 has been suggested to regulate CK2 holoenzyme polymerization [41] and is mediated by crucial cell cycle kinase CDK1 [42, 43]. Western blot confirmed the decrease of phosphorylation at Ser-209 of CK2β in LAT1-inhibited cells (Fig. 4D).

### Evaluation of antiproliferation effects by the combination of JPH203 with CK2 inhibitor

**Fig. 5:**
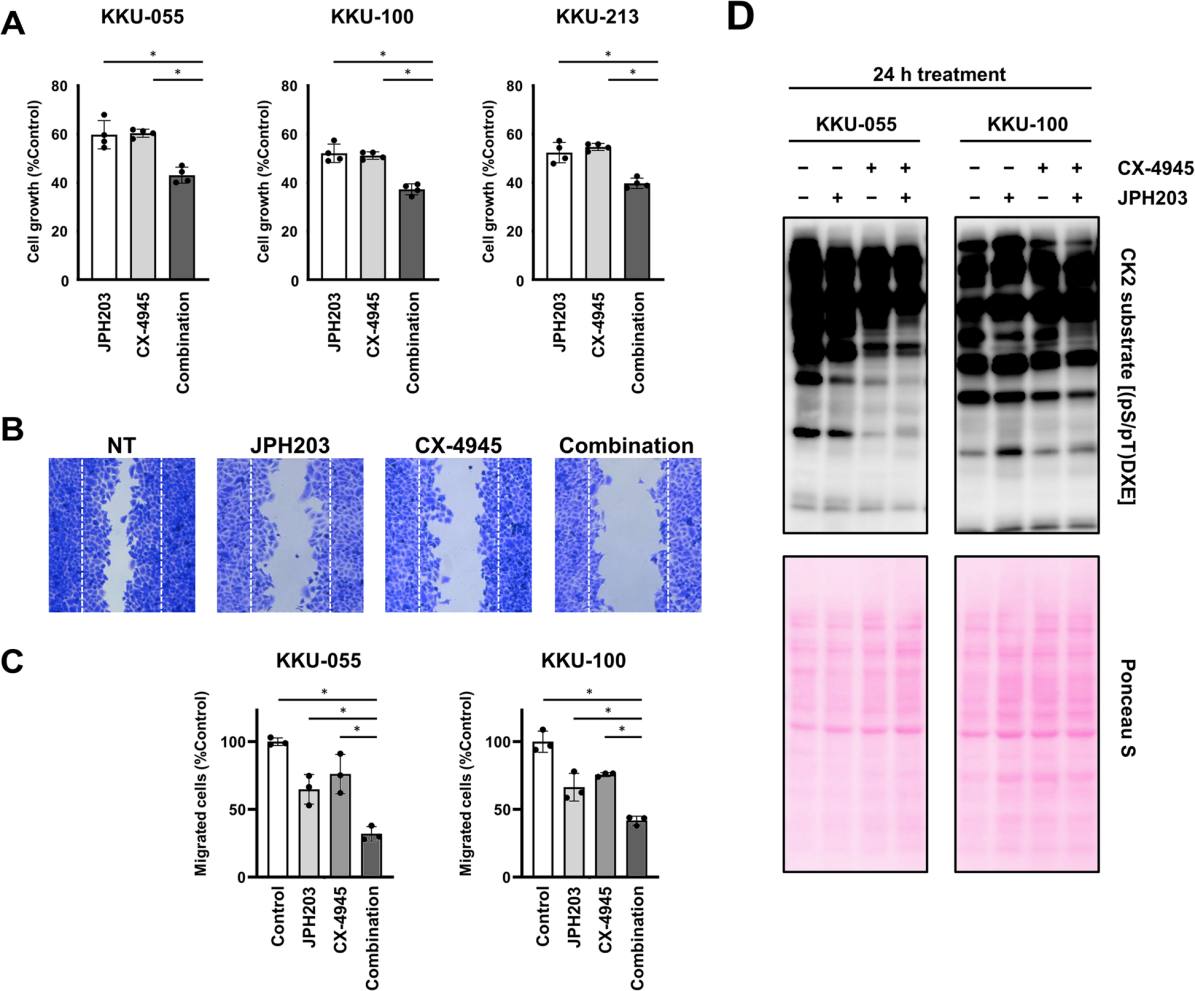
Evaluation of the combined treatment of JPH203 and CX-4945. **A** Effect of the combination of JPH203 with CX-4945 on cell proliferation. BTC cell lines were treated with JPH203 or CX-4945 alone, or in combination. After 3 days of treatment, cell growth was assessed by WST assay. The absorbance at 450 nm of each sample was expressed as % of the control without JPH203 treatment. **B** Wound healing assay using KKU-100 treated with 30 μM JPH203 or 5μM CX-4945 alone, or in combination. **C** The number of migrating cells of KKU-055 and KKU-100 treated with 30 μM JPH203 or 5 μM CX-4945 alone, or in combination. The significance of the difference between the treatment with each inhibitor alone and the combination was determined by one-way ANOVA followed by Tukey’s multiple comparison test. Data represent means ± SD (*n* = 4 for WST assay, *n* = 3 for wound healing assay). *Tukey post-hoc *p* value < 0.05. **D** Decreased phosphorylation of part of CK2 substrates by LAT1 inhibition with 30 μM JPH203 and 1 μM CX-4945, or in combination for 24 h. Phosphorylation on the consensus CK2 substrate motif was detected by a specific antibody

Our study showed that phosphorylation of CK2 substrates was affected by the JPH203 treatment (Fig. 4C). CK2 regulates various biological processes, including cell cycle [27]. In addition, LAT1 inhibition with JPH203 treatment induces G1 arrest in BTC cell lines [26]. LAT1 inhibitor and CK2 inhibitor have antiproliferation effects and are currently in clinical trials for cancer treatment [16, 27]. We, therefore, proposed that the inhibition of CK2 could enhance the antiproliferation effect induced by LAT1 inhibition. We evaluated the effect of a combination of JPH203 with CK2-selective inhibitor CX-4945 [44]. The combination significantly reduced the proliferation of all three BTC cell lines compared with JPH203 or CX-4945 alone (Fig. 5A).

Furthermore, previous studies have shown that JPH203 [18, 21] and CX-4945 [27] suppress cell migration. We, therefore, examined the effect of the combination of these two drugs on the migration of BTC cell lines. Wound healing assay showed that this combination significantly augmented cell migration suppression in KKU-055 and KKU-100 compared with each drug alone (Fig. 5B, C). Western blot showed that phosphorylation of CK2 substrates was reduced efficiently by the combination, compared with JPH203 and CX-4945 alone (Fig. 5D).

## Discussion

Our present study demonstrates changes in the phosphoproteome induced by short-time LAT1 inhibition. It has been shown that LAT1 substrates are involved in cellular signaling [3, 20], and the transport of leucine, a substrate of LAT1, is greatly decreased by LAT1 inhibition in cancer cells [8, 10, 11, 14, 15, 17–19]. This suggests that there may be components of the phosphorylation signal that depends on continuous stimulation by intracellular amino acids maintained by LAT1. We studied the overall changes in protein phosphorylation due to LAT1 inhibition using a specific LAT1 inhibitor, JPH203. Short-time JPH203 treatment was employed to reduce the possible influence of homeostatic compensatory cellular responses on phosphorylation signals associated with prolonged treatment. Our present study revealed phosphorylation sites, signaling cascades, and possible regulators immediately responding to LAT1 inhibition. The possible regulators include kinases and a phosphatase inhibitor commonly inactivated in different cell lines (Fig. 3A). The common inactivation of these was not evident in our previous proteomics study of prolonged JPH203 treatment [26], suggesting the usefulness of phosphoproteomics with short-time inhibitor treatment to reveal changes in phosphorylation that would be masked by the homeostatic compensatory cellular response in prolonged LAT1 inhibition.

This study showed substantial contributions of LAT1 to maintaining amino acid pools in BTC cells. LAT1 is highly expressed in cancer cells and is a candidate target for cancer treatment [3]. Previous studies have shown that LAT1 inhibitors inhibit amino acid transport activity in cancer cells [8, 10, 11, 14, 15, 17–19]. However, to what extent LAT1 contributes to maintaining amino acid pools in cancer cells remains unclear. The present study found that JPH203 treatment greatly decreased seven of eight LAT1 substrates in BTC cell lines within 30 min (Fig. 1), which indicates that maintenance of these amino acids is highly dependent on the uptake mediated by LAT1. Of the seven LAT1 substrates, six are essential amino acids: isoleucine, tryptophan, valine, histidine, phenylalanine, and leucine, which demonstrates the physiological importance of LAT1 in cancer cells. Methionine is a single LAT1 substrate that was not decreased by LAT1 inhibition. This may be due to the dependence of the intracellular methionine level on other transporters or unknown compensatory mechanisms.

We revealed the influence of short-time LAT1 inhibition on protein phosphorylation in BTC cells. Our phosphoproteomics identified hundreds to thousands of differentially phosphorylated sites in cells treated with JPH203 for 15 and 30 min. Upstream regulator analysis of proteins with differentially phosphorylated sites suggested the inactivation of mTOR (Fig. 3A), which was confirmed by Western blot (Fig. 4A). Since leucine was decreased in cells treated with JPH203 within 30 min (Fig. 1), mTORC1 inactivation caused by short-time LAT1 inhibition is consistent with a well-known mechanism that leucine stimulates mTORC1 [45]. LAT1 inhibition by JPH203 treatment has been reported to suppress mTOR in cancer cells [12, 13, 15], and targeting LAT1 have been suggested to be synergized with rapamycin for cancer therapy [12]. However, in our previous proteomics study, the suppression of mTOR signaling was not evident with prolonged LAT1 inhibition [26], which may be due to the technical limitations of proteomic methods. These further supports the importance of short-time treatment to avoid compensatory cellular responses.

Our previous proteomics study on BTC cells with prolonged JPH203 treatment (24 h) showed the influence of LAT1 inhibition on the cell cycle, especially involving CDK1 and CDK2 [26]. In the present study with short-time JPH203 treatment, upstream regulator analysis suggested the inactivation of cell cycle-related kinases and proteins, including CDK1 (Table S4). However, the inactivation of the cell cycle-related proteins was suggested only in KKU-055. In addition, although decreased intracellular amino acid levels were shown in all three cell lines (Fig. 1), the inactivation of signals stimulated by amino acids and leucine was suggested only in KKU-100 (Table S4). Therefore, the influence of LAT1 inhibition on the cell cycle and amino acid starvation signal response seems to appear in a temporally distinct manner among cell lines. The time dependence of individual phosphorylation responses to LAT1 inhibition and its underlying mechanism remains unclear and will focus on future research.

Our phosphoproteomics illustrated that LAT1 inhibition decreased CK2-mediated phosphorylation. The present study showed the commonly downregulated phosphorylation of proteins involved in the cell cycle and RNA splicing, and proteins of chromosome components in LAT1-inhibited cells (Fig. 3B, C). CK2α, one of the commonly downregulated regulators suggested in our phosphoproteomics (Fig. 3A), is the catalytic subunit of CK2. CK2 is a ubiquitous pro-survival protein kinase with hundreds of substrate proteins involved in cellular processes such as cell cycle and RNA splicing [27–30]. In addition, the previous study has reported enrichment of CK2 substrate proteins involved in chromosome condensation, and its functions [31]. Therefore, we focused on CK2, and found a decrease in phosphorylation of part of CK2 substrates in LAT1-inhibited cells (Fig. 4C). Not every CK2 substrate decreased phosphorylation, suggesting a change in substrate protein selectivity rather than a decrease in CK2 catalytic activity.

Due to its complexity, the regulatory mechanism by which CK2 phosphorylates hundreds of substrates is still debated. However, some studies have reported the regulatory mechanism of CK2 through modulation of the selectivity of substrate proteins by its interacting proteins and post-translational modifications [39, 40, 46]. Pin1 inhibits CK2 phosphorylation of TOP2A [36]. NOLC1, one of the CK2 substrates [47], also inhibits phosphorylation of α-casein by CK2 [39, 40]. In addition, phosphorylation of CK2α enhances its interaction with Pin1 [46]. Phosphorylation and glycosylation of CK2α alter substrate protein selectivity without significant changes in kinase activity with synthetic peptide substrate [46]. In our study, phosphorylation of CK2 substrates was decreased by LAT1 inhibition without a decrease in expression of CK2 subunits nor the kinase activity against synthetic peptides (Fig. 4B, C, and E). This suggests that LAT1 inhibition regulates substrate protein selectivity of CK2.

Although the detailed mechanism that regulates CK2 phosphorylation by LAT1 inhibition remains unknown, it should be noted that JPH203 treatment decreased phosphorylation of several sites of NOLC1 (Table S9), a CK2-regulating protein whose binding to CK2 is influenced by phosphorylation [39, 40]. Our study revealed that CK2-NOLC1 interaction was abolished by LAT1inhibition (Fig. 4F), which indicates that LAT1 inhibition regulates CK2 function by interrupting its interaction with NOLC1. Since previous studies utilized α-casein as a substrate of CK2 [39, 40], further studies are needed to identify proteomes whose CK2-mediated phosphorylation is influenced by CK2-NOLC1 interaction changed by LAT1 inhibition. Furthermore, JPH203 treatment also decreased phosphorylation of Ser-209 of CK2β (Fig. 4D and Table S9), a regulatory subunit of CK2 that can regulate CK2α substrate specificity [38]. In addition, it has been suggested that phosphorylation of Ser-209 of CK2β plays a role in polymeric assemblies of CK2 holoenzyme [41]. An upstream kinase that phosphorylates Ser-209 is CDK1 [43, 48]. Previously, we have shown the significant involvement of CDK1 in LAT1 inhibitor-mediated effects [26]. These findings may provide clues for further study to investigate the mechanism regulating CK2 function by LAT1 inhibition. In addition, although we showed both JPH203 and BCH decreased phosphorylation of CK2 substrates, it remains unknown whether these effects induced by LAT1 inhibition are due to a decrease of specific amino acids or global essential amino acids. Further studies on each amino acid's depletion or combination are needed.

Our phosphoproteomics suggested that short-time JPH203 treatment affects CK2 phosphorylation. CK2 phosphorylates a subset of its substrates in response to many cellular stimuli, such as heat shock, UV irradiation, hypoxia, and viral infections [49]. Recently, low glucose has been reported to decrease CK2 phosphorylation by inhibiting CK2-substrate complex formation [50]. In the present study, phosphorylation of CK2 substrate, Ser-1469 of TOP2A, was decreased by short-time treatment of JPH203 or BCH. Whether the decreased CK2 phosphorylation is due to a novel mechanism that directly senses amino acids similarly to mTOR, or indirect changes signaling is a challenging future research question. By using co-immunoprecipitation assay, we found CK2-NOLC1 interaction was not abolished in short-time LAT1-inhibited cells (data not shown), which demonstrates that CK2 regulation mechanism is different for the short- and long-time treatments of JPH203.

We showed the potential of combining LAT1 inhibitor with CK2 inhibitor for cancer treatment. Abnormally high CK2 activity has been found in tumors, and CK2 inhibitors show an anticancer effect [27]. Our present study evaluated the effect of the combinations of JPH203 with CK2 inhibitor, CX-4945, and showed the reduction of cell proliferation and migration (Fig. 5). Because both JPH203 and CX-4945 are now in the clinical trials [16, 27], our research could suggest potential future indications of these drugs.

In summary, in the present study, we conducted phosphoproteomics to investigate phosphorylation signals responding to the decrease in intracellular amino acids by short-time LAT1 inhibition in BTC cells. Bioinformatics analysis of differentially phosphorylated sites revealed possible regulators responsible for the changes caused by LAT1 inhibition, including kinases and a phosphatase inhibitor inactivated commonly in distinct cell lines. Furthermore, commonly downregulated phosphorylation sites were detected in proteins involved in various biological processes such as cell cycle and RNA splicing. We further examined one of the possible regulators, CK2, involved in cell cycle and RNA splicing. We revealed downregulation of phosphorylation of CK2 substrates and CK2 regulatory proteins, and abolished interaction between CK2 and its regulatory protein NOLC1 in LAT1-inhibited cells. Moreover, a combination of LAT1 and CK2 inhibitors significantly inhibited the proliferation and migration of BTC cells. This study provides new insights involving CK2 in the role of LAT1 in cancer cells and the mechanisms of antitumor action of LAT1 inhibitors.

## Supplementary Information

**Additional file 1: Figure S1.** Amino acid levels of BTC cells treated with JPH203. Each amino acid amount was normalized by protein amount (pmol/μg protein): LAT1 substrates in KKU-055 (A), KKU-100 (C), and KKU-213 (E); non-LAT1 substrates in KKU-055 (B), KKU100 (D), and KKU-213 (F). **Figure S2.** Reproducibility between biological replicates of phosphoproteomics conducted on KKU-055, KKU-100, and KKU-213. Scatter plots showing log2 fold changes of phosphoproteome between biological replicates of JPH203-treated/Control samples of 15 min (A) and 30 min (B) treatment are shown in each cell line. Plots of differentially phosphorylated sites are shown in red. **Figure S3.** Changes of phosphorylation caused by LAT1 inhibition in phosphoproteomics conducted on KKU-055, KKU-100, and KKU-213 cells. Volcano plots were generated by plotting –log10 *p*-values against log2 fold changes of relative abundance ratio between JPH203-treated and control samples. Differentially phosphorylated sites are indicated in green and cyan for upregulation and downregulation, respectively. **Figure S4.** Networks of proteins with upregulated (A) and downregulated (B) phosphorylation commonly detected in phosphoproteomics. Proteins with phosphorylation commonly changed in at least two phosphoproteomics results analyzing three cell lines of BTC treated with JPH203 for 15 min and 30 min (a total of 6 results) were subject to SPRING. **Figure S5.** Changes in the phosphorylation of substrates of suggested key kinases by LAT1 inhibition. Protein extracted from cells treated with 50 mM BCH and control cells was analyzed by Western blot. The phosphorylation at Ser-1469 of TOP2A were decreased by BCH treatment KKU-055 and KKU-100 for 1 h, and KKU-213 for 30 min. **Figure S6.** Phosphorylation of CK2 substrates of BTC cells treated with JPH203 for 15 and 30 min in Western blot. Phosphorylation on the consensus CK2 substrate motif was detected by a specific antibody. **Figure S7.** CK2 activity of BTC cells treated with JPH203. The extracted protein of BTC cells treated with 30 μM JPH203 for 15 min was subject to CK2 activity assay by ELISA using p53 N-terminal peptide and p53-pS46 antibody conjugated with horseradish peroxidase.

**Additional file 2: Table S1.** TMT labeling scheme for phosphoproteomics.

**Additional file 3: Table S2.** Quantitative phosphopeptides identified in three BTC cell lines.

**Additional file 4: Table S3.** Quantitative phosphorylation sites identified in three BTC cell lines.

**Additional file 5: Table S4.** Possible upstream regulators associated with LAT1 inhibition by JPH203 treatment.

**Additional file 6: Table S5.** Possible upstream regulators associated with LAT1 inhibition by JPH203 treatment which are commonly detected in different cell lines.

**Additional file 7: Table S6.** Canonical pathways associated with LAT1 inhibition by JPH203 treatment.

**Additional file 8: Table S7.** Phosphorylation sites commonly upregulated or downregulated in the tested phosphoproteomes.

**Additional file 9: Table S8.** Functional annotation clustering using DAVID for proteins with the upregulated and downregulated phosphorylation commonly detected in phosphoproteomes analyzed in this study.

**Additional file 10: Table S9.** Quantification results of CK2-regulatory proteins in previous phosphoproteomics and proteomics of BTC cells with JPH203 for 24 h.

## Data Availability

The datasets used and/or analyzed during the current study are available from the corresponding author on reasonable request.

## Abbreviations

BCH: 2-amino-2-norbornane carboxylic acid
BTC: Biliary tract cancer
FBS: Fetal bovine serum
GO: Gene Ontology
IMAC: Immobilized metal affinity chromatography
IPA: Ingenuity pathway analysis
mTORC1: Mechanistic target of rapamycin kinase complex 1
LAT1: l-type amino acid transporter 1
LC-MS/MS: Liquid chromatography-tandem mass spectrometry
TFA: Trifluoroacetic acid
TMT: Tandem mass tag
WST: Water-soluble tetrazolium
