# Supplementary material for: Phosphoproteomics revealed cellular signals immediately responding to disruption of cancer amino acid homeostasis induced by inhibition of l-type amino acid transporter 1
Source: Cancer Metab. 2022 Nov 10;10:18. doi: 10.1186/s40170-022-00295-8 (PMC9650822; doi:10.1186/s40170-022-00295-8)
Supplement: Supplementary file 1 — Additional file 1: Figure S1. Amino acid levels of BTC cells treated with JPH203. Each amino acid amount was normalized by protein amount (pmol/μg protein): LAT1 substrates in KKU-055 (A), KKU-100 (C), and KKU-213 (E); non-LAT1 substrates in KKU-055 (B), KKU100 (D), and KKU-213 (F). Figure S2. Reproducibility between biological replicates of phosphoproteomics conducted on KKU-055, KKU-100, and KKU-213. Scatter plots showing log2 fold changes of phosphoproteome between biological replicates of JPH203-treated/Control samples of 15 min (A) and 30 min (B) treatment are shown in each cell line. Plots of differentially phosphorylated sites are shown in red. Figure S3. Changes of phosphorylation caused by LAT1 inhibition in phosphoproteomics conducted on KKU-055, KKU-100, and KKU-213 cells. Volcano plots were generated by plotting –log10 p-values against log2 fold changes of relative abundance ratio between JPH203-treated and control samples. Differentially phosphorylated sites are indicated in green and cyan for upregulation and downregulation, respectively. Figure S4. Networks of proteins with upregulated (A) and downregulated (B) phosphorylation commonly detected in phosphoproteomics. Proteins with phosphorylation commonly changed in at least two phosphoproteomics results analyzing three cell lines of BTC treated with JPH203 for 15 min and 30 min (a total of 6 results) were subject to SPRING. Figure S5. Changes in the phosphorylation of substrates of suggested key kinases by LAT1 inhibition. Protein extracted from cells treated with 50 mM BCH and control cells was analyzed by Western blot. The phosphorylation at Ser-1469 of TOP2A were decreased by BCH treatment KKU-055 and KKU-100 for 1 h, and KKU-213 for 30 min. Figure S6. Phosphorylation of CK2 substrates of BTC cells treated with JPH203 for 15 and 30 min in Western blot. Phosphorylation on the consensus CK2 substrate motif was detected by a specific antibody. Figure S7. CK2 activity of BTC cells treated with JPH203. [file 40170_2022_295_MOESM1_ESM.pdf]

Fig. S1. Amino acid levels of BTC cells treated with JPH203. Each amino acid amount was normalized by protein amount (pmol/μg protein): LAT1 substrates in KKU-055 (A), KKU-100 (C), and KKU-213 (E); non-LAT1 substrates in KKU-055 (B), KKU-100 (D), and KKU-213 (F).

A

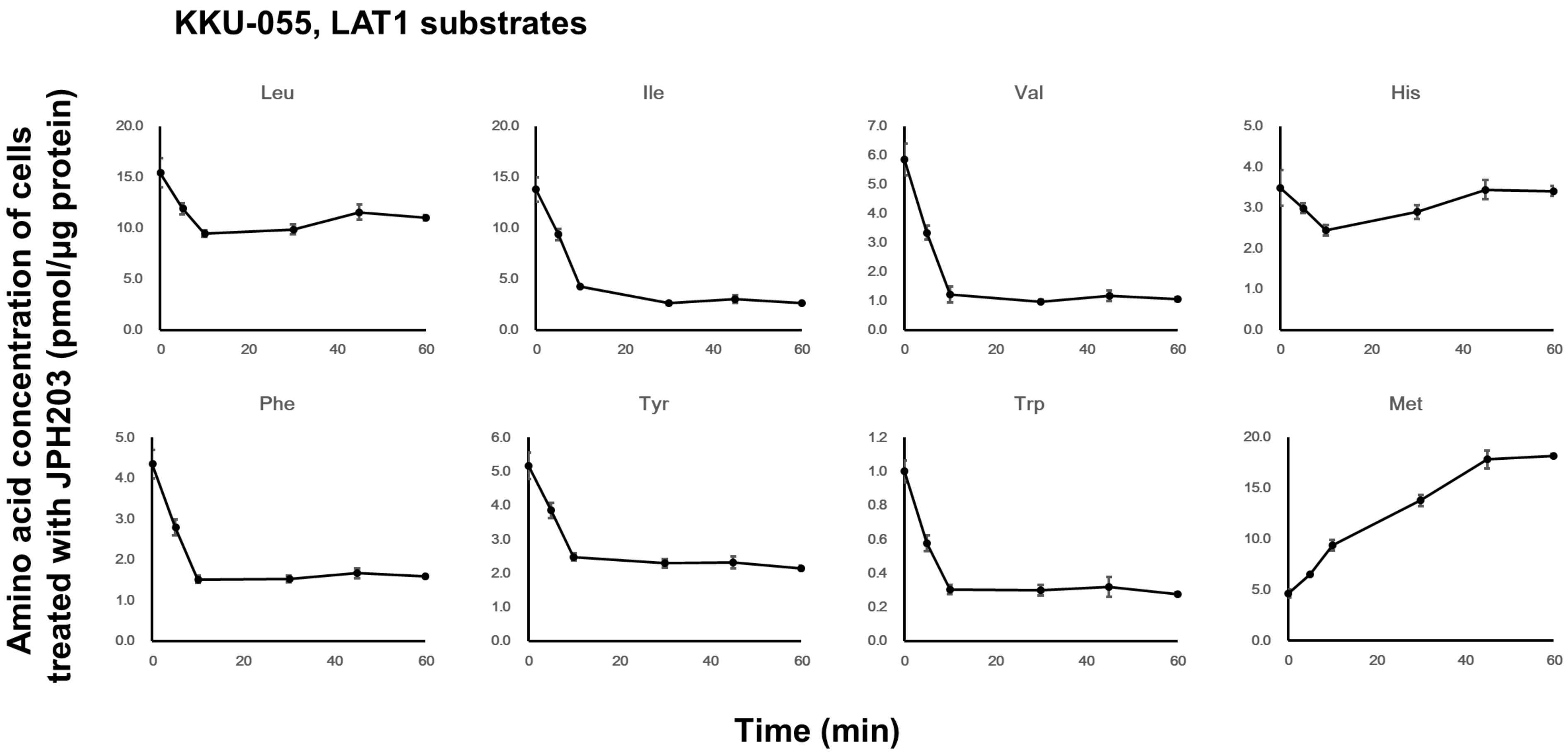
KKU-055, LAT1 substrates
Leu
Ile
Val
His
Phe
Tyr
Trp
Met
Amino acid concentration of cells treated with JPH203 (pmol/μg protein)
Time (min)

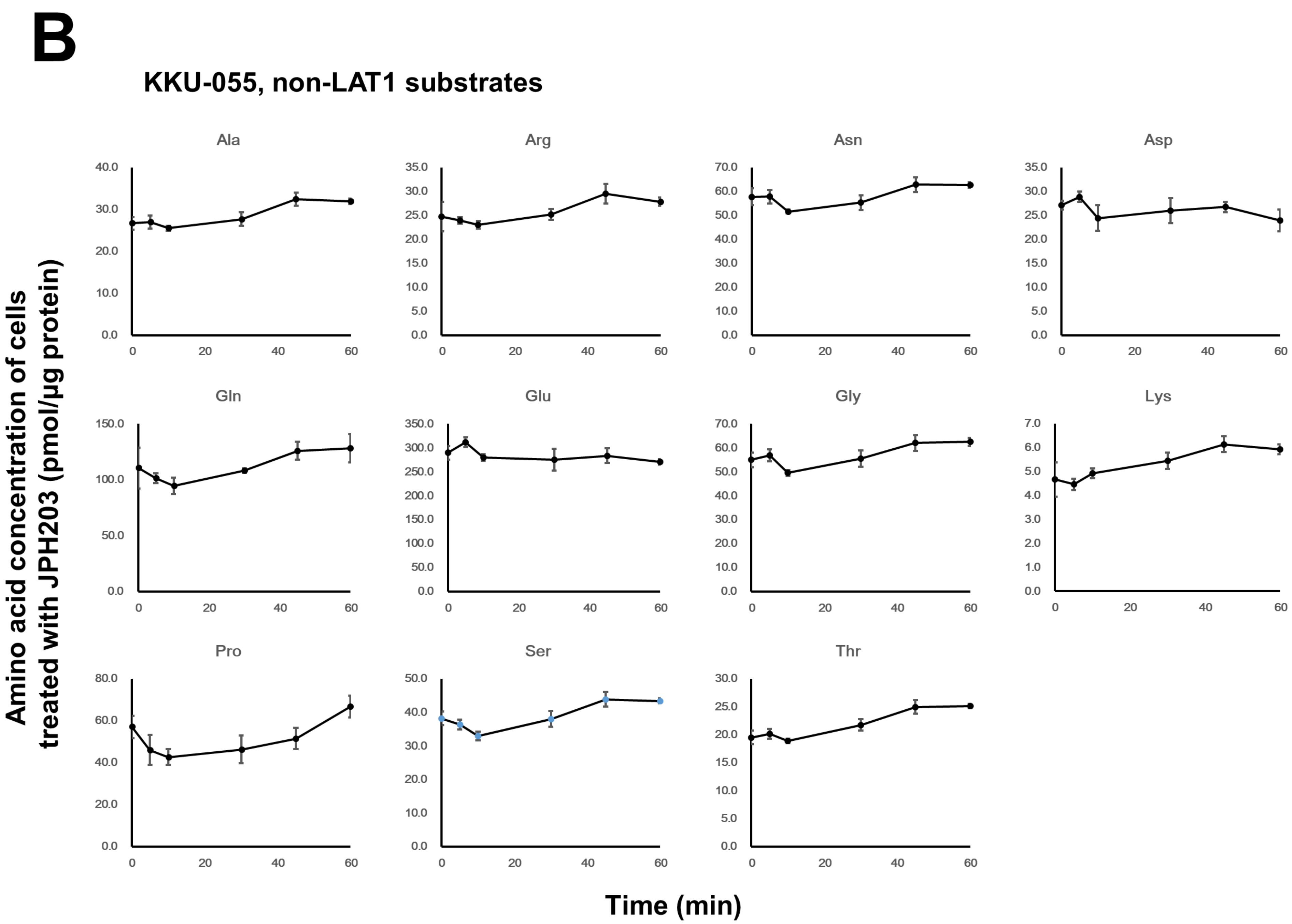
B
KKU-055, non-LAT1 substrates
Ala
Arg
Asn
Asp
Gln
Glu
Gly
Lys
Pro
Ser
Thr
Amino acid concentration of cells treated with JPH203 (pmol/µg protein)
Time (min)

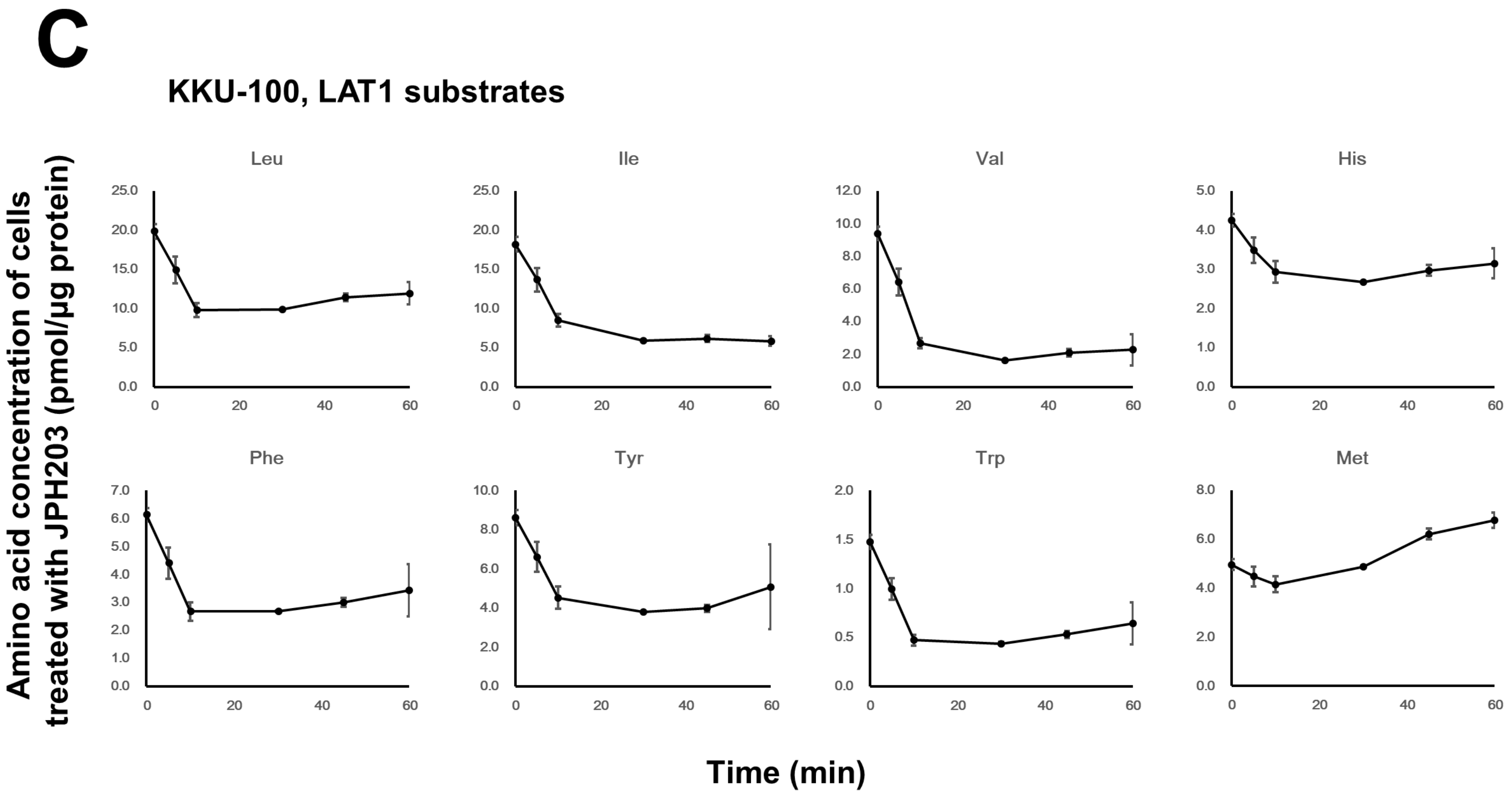
C
KKU-100, LAT1 substrates
Leu
Ile
Val
His
Phe
Tyr
Trp
Met
Amino acid concentration of cells treated with JPH203 (pmol/µg protein)
Time (min)

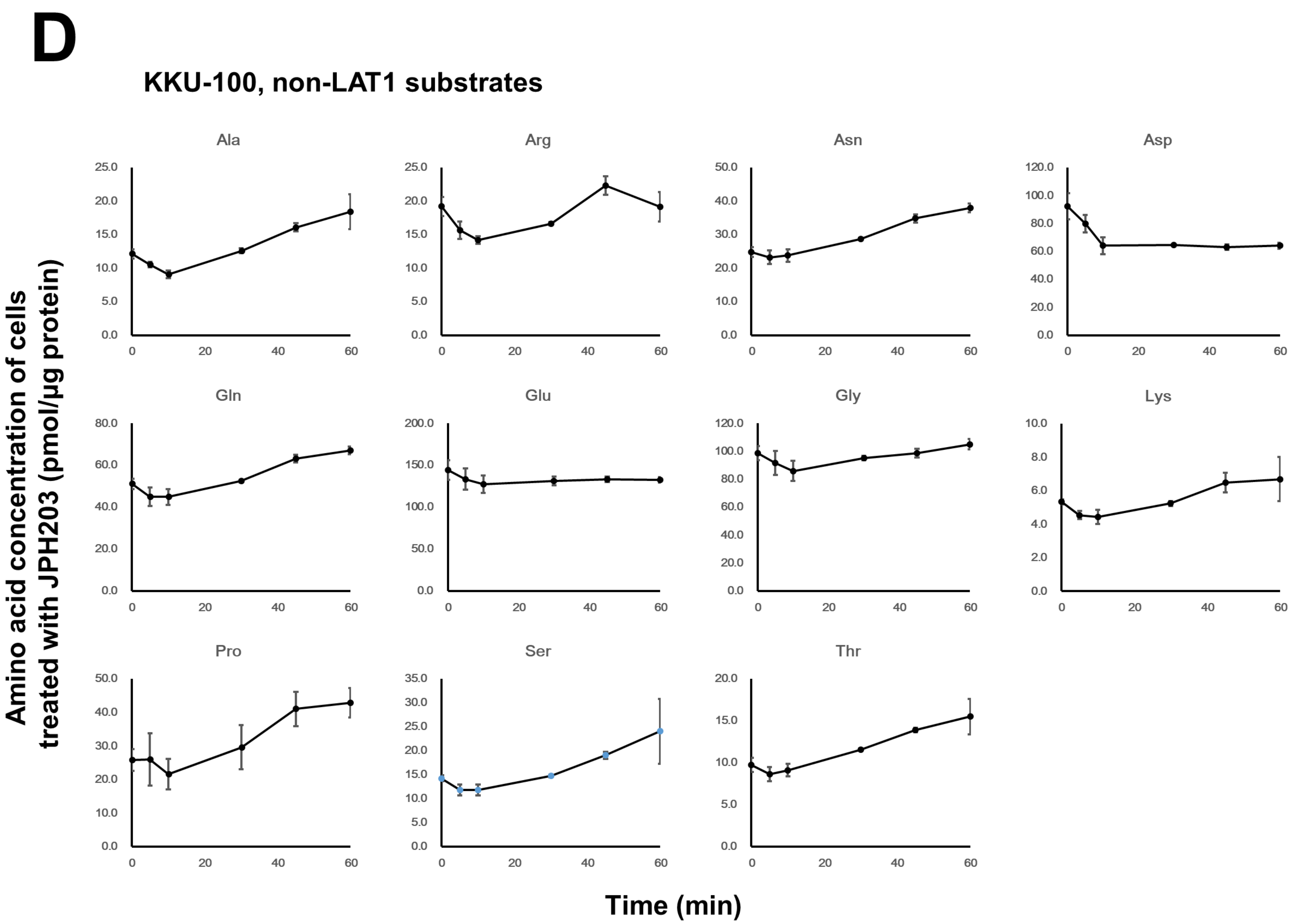
D
KKU-100, non-LAT1 substrates
Ala
Arg
Asn
Asp
Gln
Glu
Gly
Lys
Pro
Ser
Thr
Amino acid concentration of cells treated with JPH203 (pmol/μg protein)
Time (min)

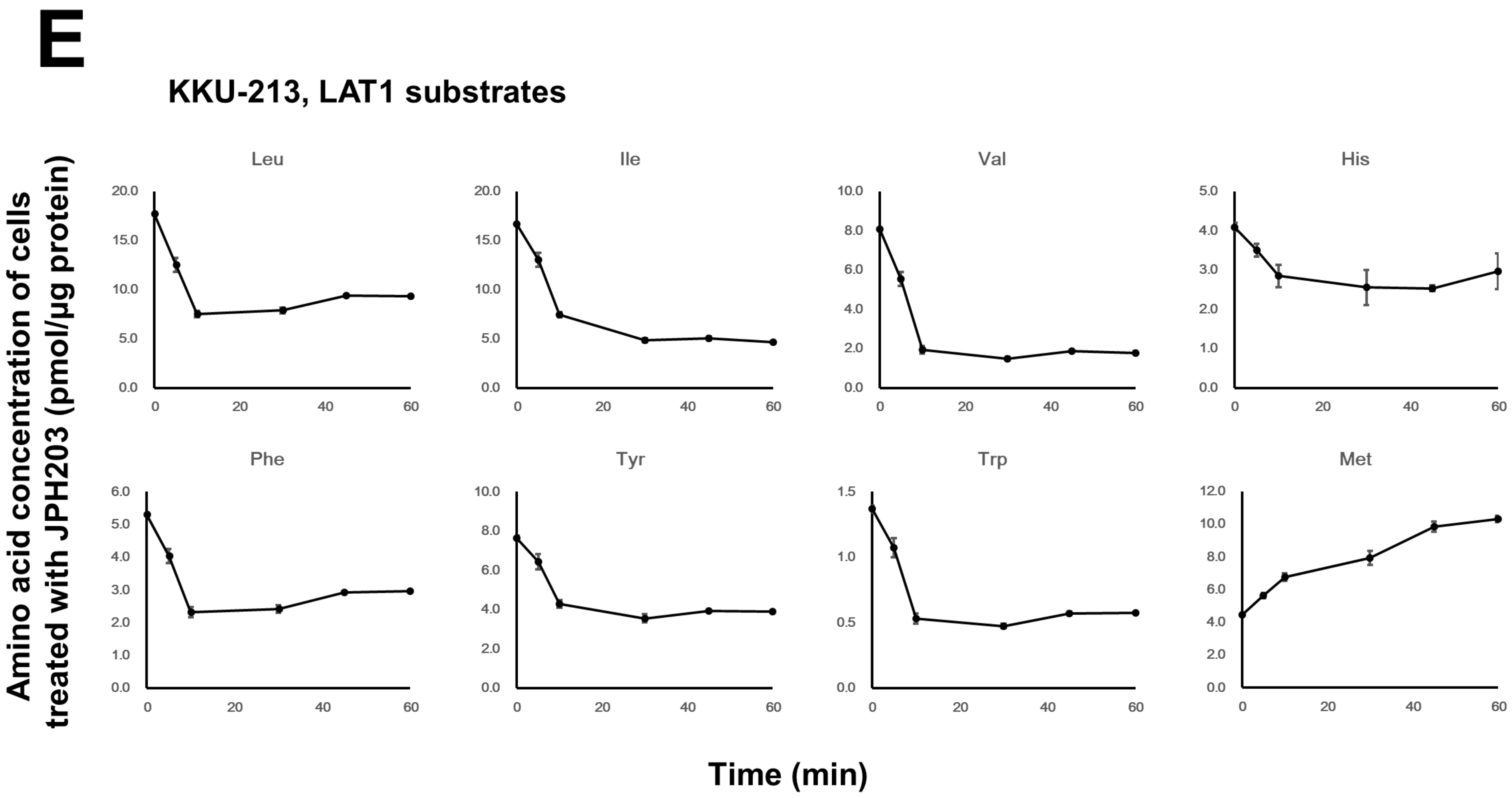
E
KKU-213, LAT1 substrates
Leu
Ile
Val
His
Phe
Tyr
Trp
Met
Amino acid concentration of cells treated with JPH203 (pmol/µg protein)
Time (min)

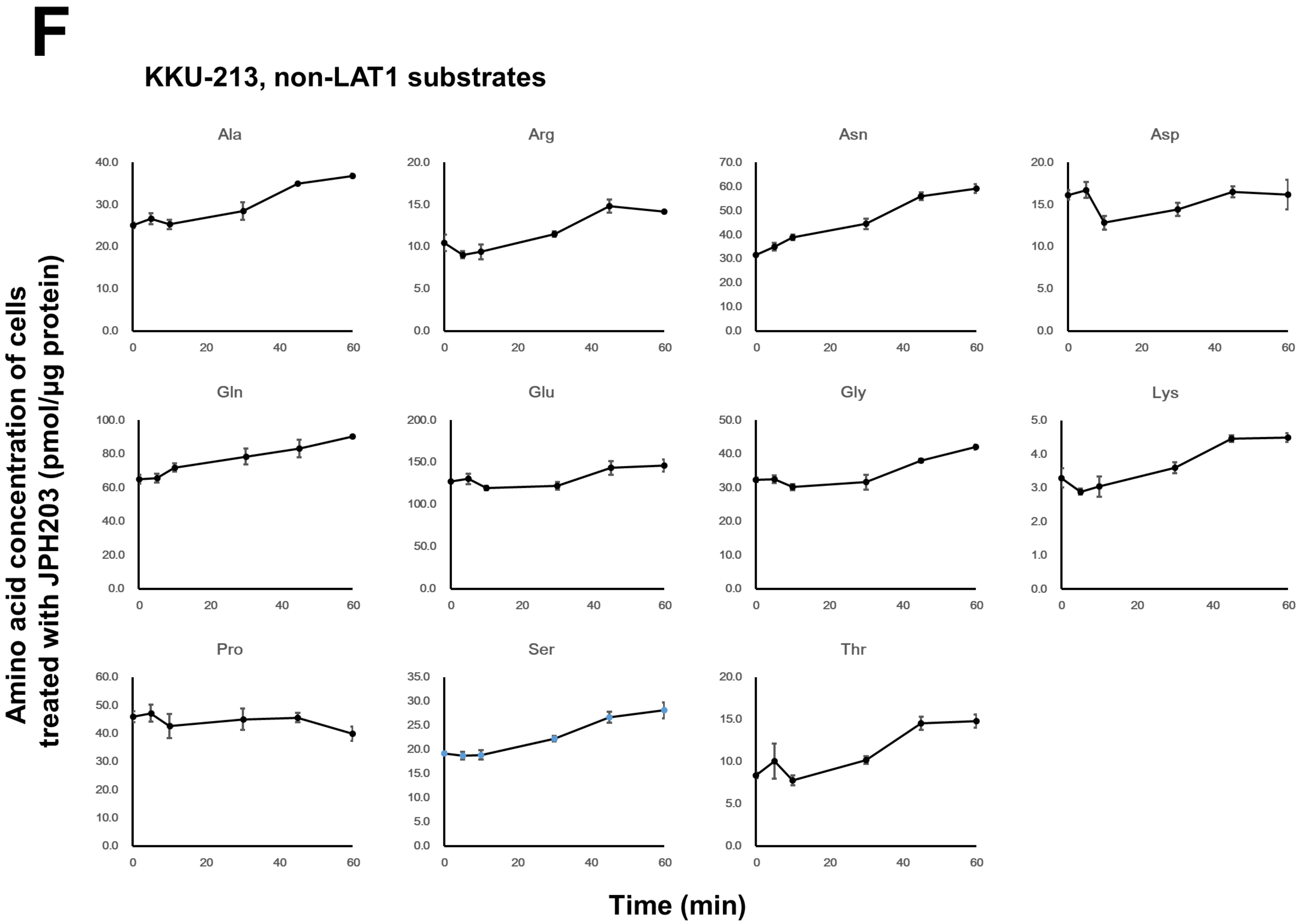
F
KKU-213, non-LAT1 substrates
Ala
Arg
Asn
Asp
Gln
Glu
Gly
Lys
Pro
Ser
Thr
Amino acid concentration of cells treated with JPH203 (pmol/μg protein)
Time (min)

Fig. S2. Reproducibility between biological replicates of phosphoproteomics conducted on KKU-055, KKU-100, and KKU-213. Scatter plots showing log2 fold changes of phosphoproteome between biological replicates of JPH203-treated/Control samples of 15 min (A) and 30 min (B) treatment are shown in each cell line. Plots of differentially phosphorylated sites are shown in red.

A

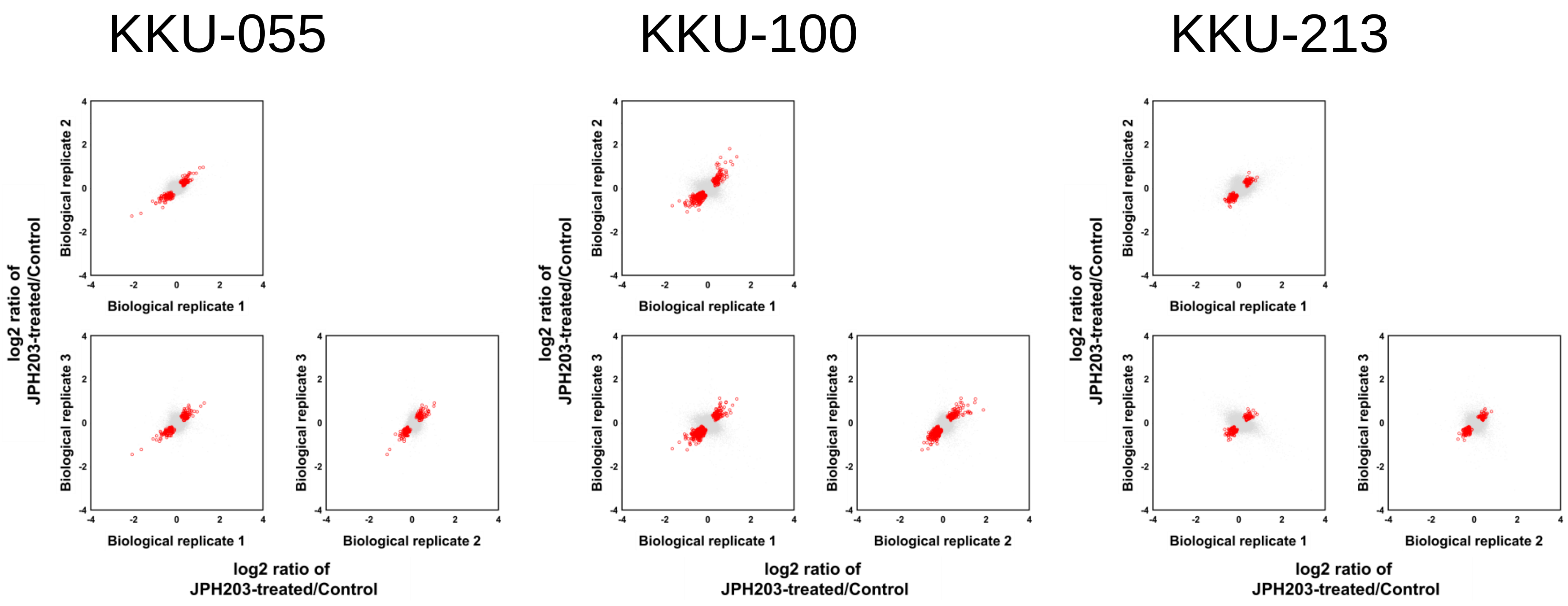

JPH203 treatment, 15 min

B

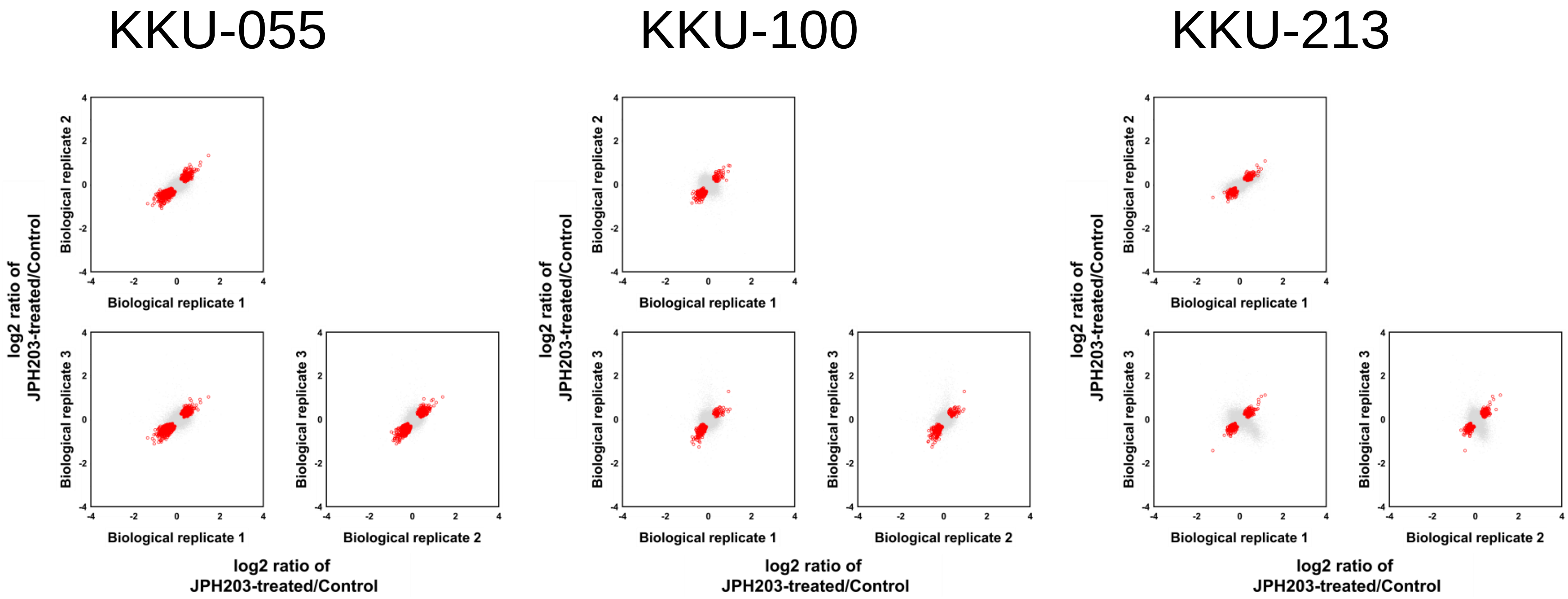

JPH203 treatment, 30 min

Fig. S3. Changes of phosphorylation caused by LAT1 inhibition in phosphoproteomics conducted on KKU-055, KKU-100, and KKU-213 cells. Volcano plots were generated by plotting –log10 *p*-values against log2 fold changes of relative abundance ratio between JPH203-treated and control samples. Differentially phosphorylated sites are indicated in green and cyan for upregulation and downregulation, respectively.

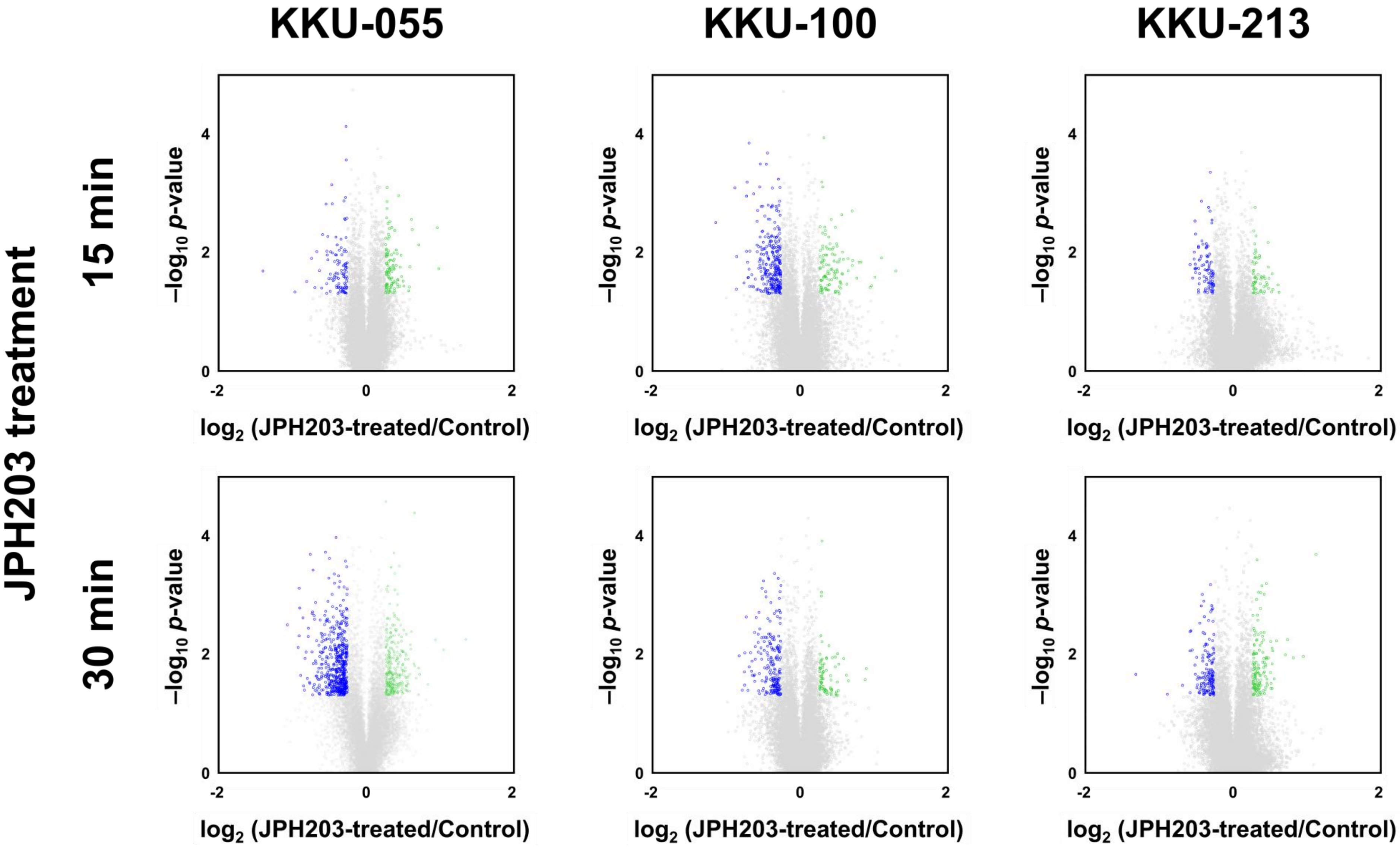

JPH203 treatment
15 min
30 min
KKU-055
KKU-100
KKU-213
−log10 p-value
log2 (JPH203-treated/Control)
0
2
4
-2
0
2

Fig. S4. Networks of proteins with upregulated (A) and downregulated (B) phosphorylation commonly detected in phosphoproteomics. Proteins with phosphorylation commonly changed in at least two phosphoproteomics results analyzing three cell lines of BTC treated with JPH203 for 15 min and 30 min (a total of 6 results) were subject to SPRING.

A

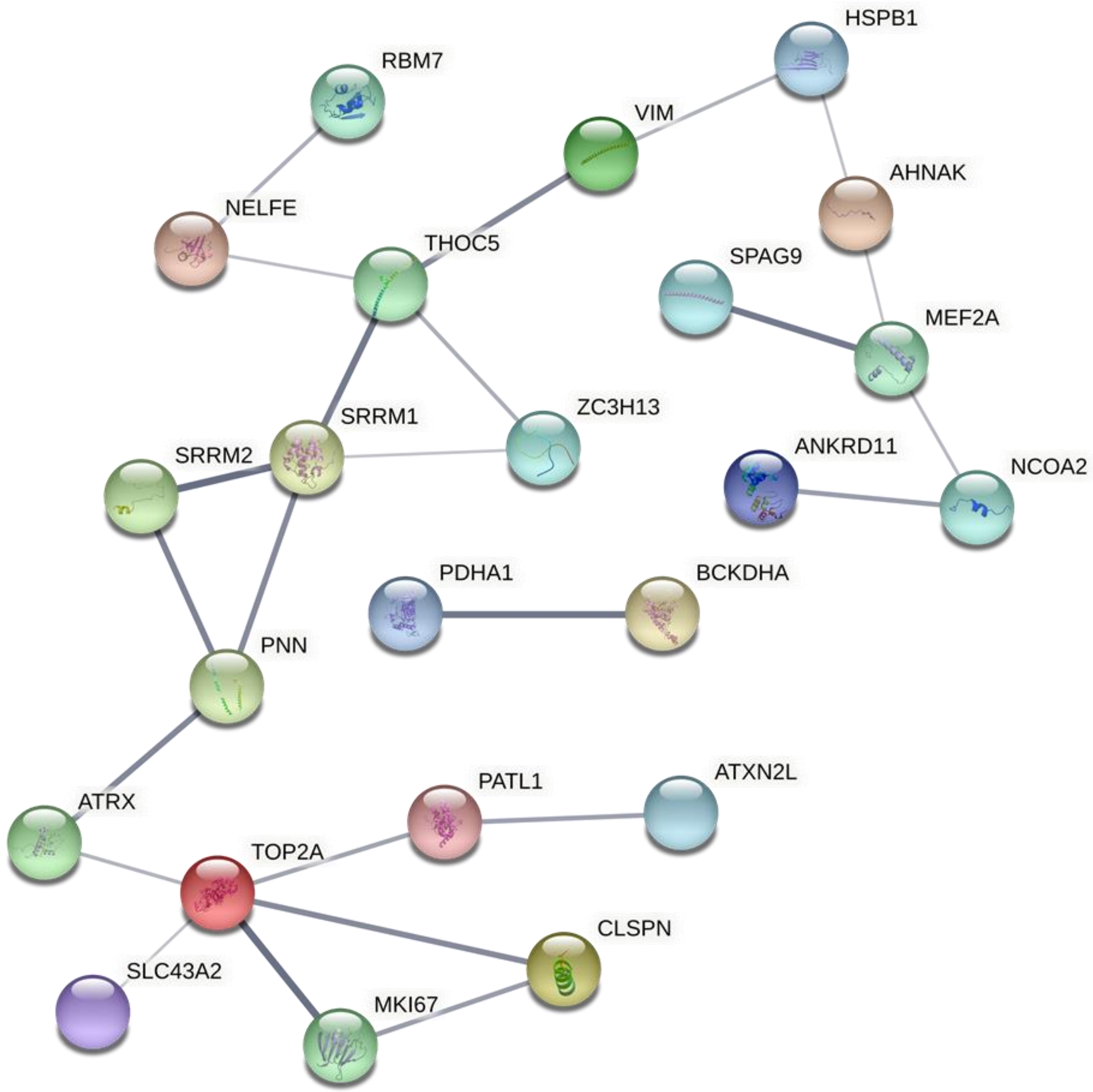

HSPB1
RBM7
VIM
AHNAK
NELFE
THOC5
SPAG9
MEF2A
ZC3H13
SRRM1
ANKRD11
SRRM2
NCOA2
PDHA1
BCKDHA
PNN
PATL1
ATXN2L
ATRX
TOP2A
CLSPN
SLC43A2
MKI67

B

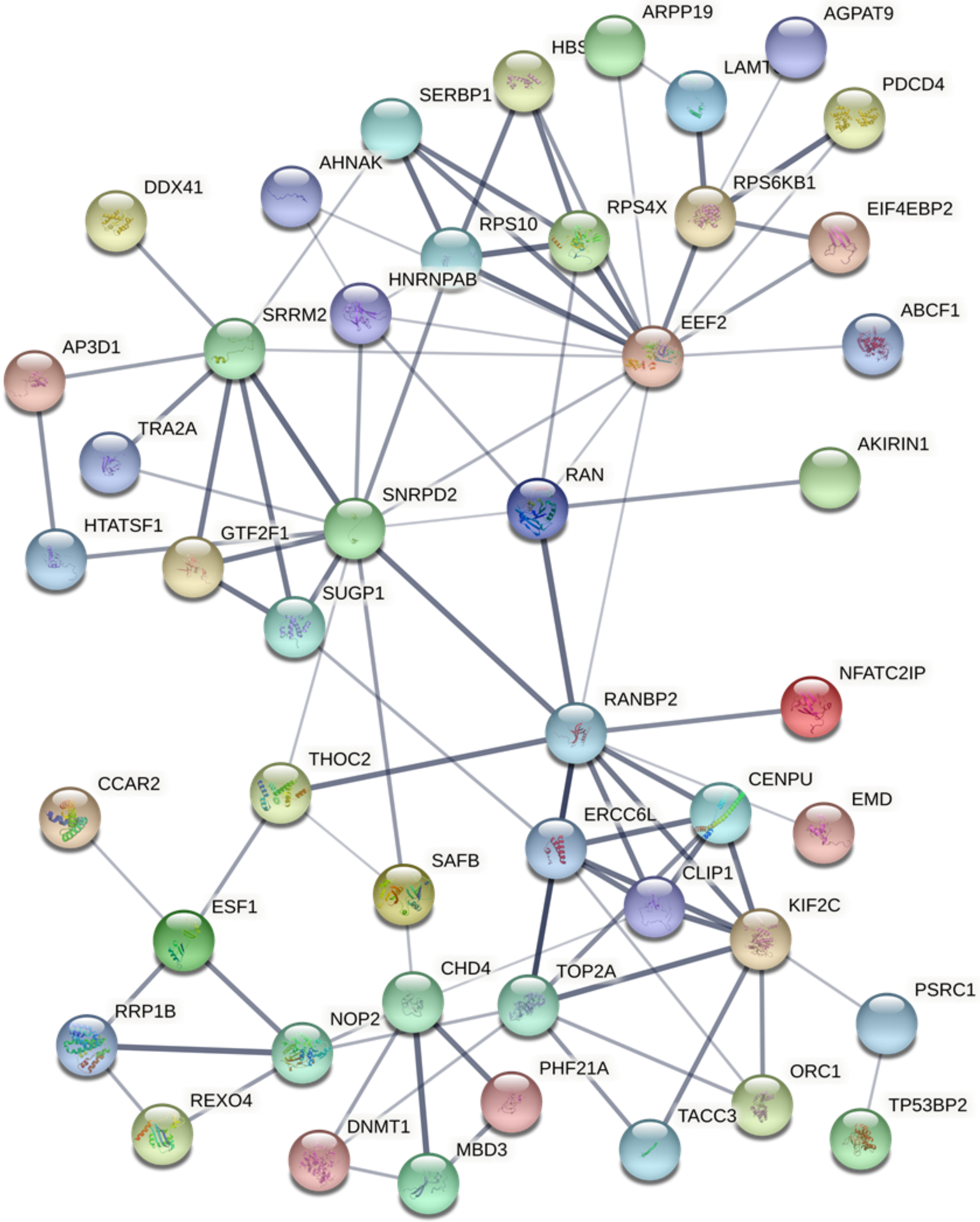

ARPP19
AGPAT9
HBS
LAMT
SERBP1
PDCD4
AHNAK
DDX41
RPS6KB1
RPS4X
RPS10
EIF4EBP2
HNRNPAB
SRRM2
EEF2
ABCF1
AP3D1
TRA2A
AKIRIN1
RAN
SNRPD2
HTATSF1
GTF2F1
SUGP1
NFATC2IP
RANBP2
THOC2
CCAR2
CENPU
EMD
ERCC6L
SAFB
CLIP1
KIF2C
ESF1
CHD4
TOP2A
PSRC1
RRP1B
NOP2
PHF21A
ORC1
REXO4
TACC3
TP53BP2
DNMT1
MBD3

Fig. S5. Changes in the phosphorylation of substrates of suggested key kinases by LAT1 inhibition. Protein extracted from cells treated with 50 mM BCH and control cells was analyzed by Western blot. The phosphorylation at Ser-1469 of TOP2A were decreased by BCH treatment KKU-055 and KKU-100 for 1 h, and KKU-213 for 30 min.

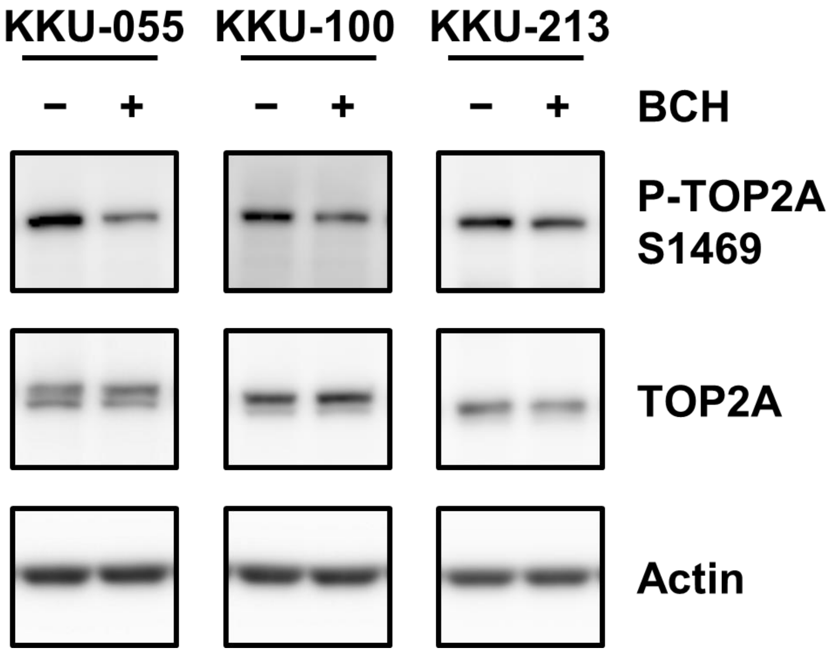
KKU-055
KKU-100
KKU-213
−
+
−
+
−
+
BCH
P-TOP2A
S1469
TOP2A
Actin

Fig. S6. Phosphorylation of CK2 substrates of BTC cells treated with JPH203 for 15 and 30 min in Western blot. Phosphorylation on the consensus CK2 substrate motif was detected by a specific antibody.

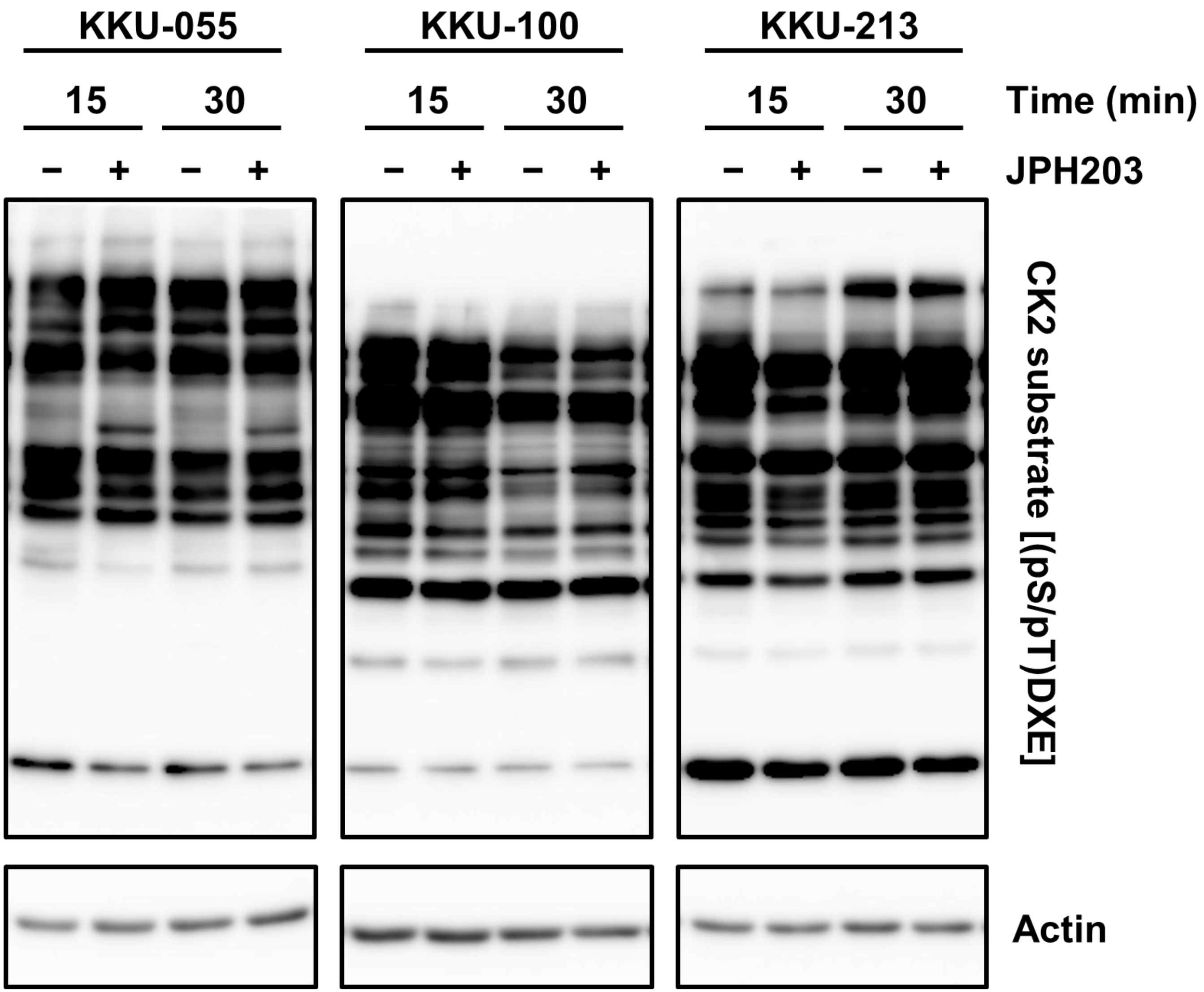

KKU-055
KKU-100
KKU-213
15
30
15
30
15
30
Time (min)
− + − +
− + − +
− + − +
JPH203
CK2 substrate [(pS/pT)DXE]
Actin

Fig. S7. CK2 activity of BTC cells treated with JPH203. The extracted protein of BTC cells treated with 30 μM JPH203 for 15 min was subject to CK2 activity assay by ELISA using p53 N-terminal peptide and p53-pS46 antibody conjugated with horseradish peroxidase.

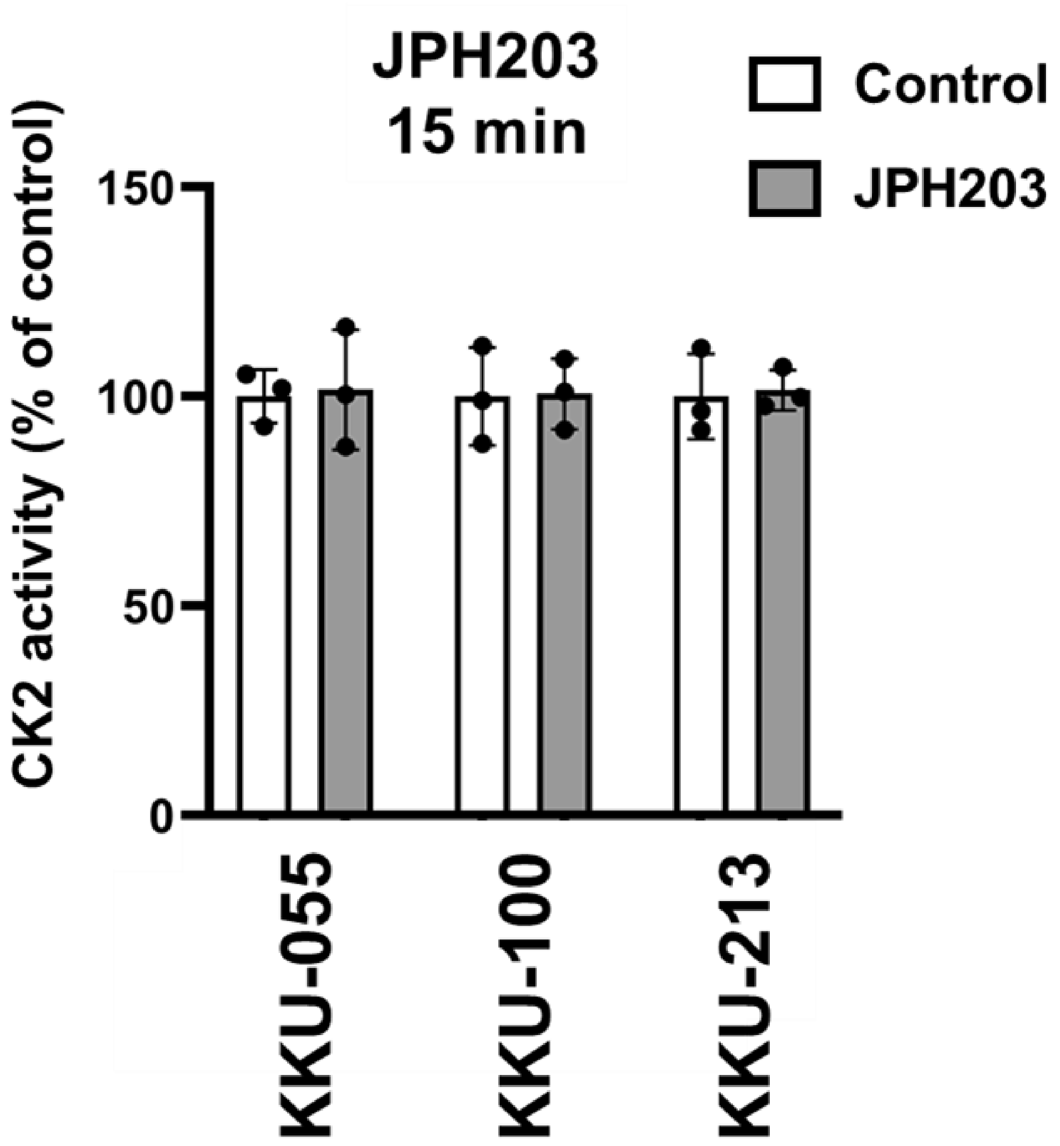
JPH203
15 min
Control
JPH203
CK2 activity (% of control)
150
100
50
0
KKU-055
KKU-100
KKU-213
